# Supplementary material for: Impact of maternity waiting homes on facility delivery among remote households in Zambia: protocol for a quasiexperimental, mixed-methods study
Source: BMJ Open. 2018 Aug 10;8(8):e022224. doi: 10.1136/bmjopen-2018-022224 (PMC6089313; doi:10.1136/bmjopen-2018-022224)
Supplement: Author's manuscript [file bmjopen-2018-022224.draft_revisions.pdf]

BMJ Open is committed to open peer review. As part of this commitment we make the peer review history of every article we publish publicly available.

When an article is published we post the peer reviewers' comments and the authors' responses online. We also post the versions of the paper that were used during peer review. These are the versions that the peer review comments apply to.

The versions of the paper that follow are the versions that were submitted during the peer review process. They are not the versions of record or the final published versions. They should not be cited or distributed as the published version of this manuscript.

BMJ Open is an open access journal and the full, final, typeset and author-corrected version of record of the manuscript is available on our site with no access controls, subscription charges or pay-per-view fees (http://bmjopen.bmj.com).

If you have any questions on BMJ Open's open peer review process please email info.bmjopen@bmj.com

BMJ Open

Impact of maternity waiting homes on facility delivery among remote households in Zambia: protocol for a quasi-experimental, mixed-methods study

Journal:	*BMJ Open*
Manuscript ID	bmjopen-2018-022224
Article Type:	Protocol
Date Submitted by the Author:	07-Feb-2018
Complete List of Authors:	Scott, Nancy; Boston University School of Public Health, Global Health<br>Kaiser, Jeanette; Boston University School of Public Health, Global Health<br>Vian, Taryn; Boston University School of Public Health, Global Health<br>Bonawitz, Rachael; Boston University School of Public Health, Global Health; Boston University School of Medicine, Pediatrics<br>Fong, Rachel; Boston University School of Public Health, Global Health<br>Ngoma, Thandiwe; Right to Care Zambia<br>Biemba, Godfrey; National Health Research Authority, Pediatric Centre of Excellence<br>Boyd, Carol; University of Michigan School of Nursing, Health Behavior & Biological Sciences<br>Lori, Jody; University of Michigan School of Nursing, Center for Global Affairs & PAHO/WHO Collaborating Center<br>Hamer, D.; Boston University,<br>Rockers, Peter; Boston University, Department of Global Health
Keywords:	maternity waiting home, maternal health, facility delivery, mixed methods, impact evaluation, Zambia

SCHOLARONE™
Manuscripts

Title: *Impact of maternity waiting homes on facility delivery among remote households in Zambia: protocol for a quasi-experimental, mixed-methods study*

Authors:

Name	Institutional Address	Email Address
Nancy Scott	Department of Global Health, Boston University School of Public Health, Boston, USA<br>801 Massachusetts Ave, Boston, MA 02118	nscott@bu.edu (corresponding)<br>Tel: +1-617-414-1278
Jeanette Kaiser	Department of Global Health, Boston University School of Public Health<br>Boston, MA USA	jlkaiser@bu.edu
Taryn Vian	Department of Global Health, Boston University School of Public Health<br>Boston, MA USA	tvian@bu.edu
Rachael Bonawitz	Department of Global Health, Boston University School of Public Health<br>Boston, MA USA<br>Department of Pediatrics, Boston University School of Medicine<br>Boston, MA USA	rebonawi@bu.edu
Rachel Fong	Department of Global Health, Boston University School of Public Health<br>Boston, MA USA	rmfong@bu.edu
Thandiwe Ngoma	Right to Care-Zambia<br>Lusaka, Zambia	ngoma.thandiwe@gmail.com
Godfrey Biemba	National Health Research Authority, Pediatric Centre of Excellence<br>Lusaka, Zambia	gbiemba@gmail.com
Carol J. Boyd	Department of Health Behavior & Biological Sciences<br>School of Nursing, University of Michigan<br>Ann Arbor, MI USA	caroboyd@med.umich.edu
Jody R. Lori	Center for Global Affairs &<br>PAHO/WHO Collaborating Center<br>School of Nursing, University of Michigan<br>Ann Arbor, MI USA	jrlori@umich.edu
Davidson H. Hamer	Department of Global Health, Boston University School of Public Health<br>Boston, MA USA<br><br>Section of Infectious Diseases, Department of Medicine, Boston Medical Center<br>Boston, MA USA	dhamer@bu.edu
Peter C. Rockers	Department of Global Health, Boston University School of Public Health<br>Boston, MA USA	prockers@bu.edu

Word count: 4,200 (excluding title page, abstract, references, figures and tables)

For peer review only - http://bmjopen.bmj.com/site/about/guidelines.xhtml

List of Acronyms

Africare/UM	Africare/University of Michigan
ANC	Antenatal care
BEmONC	Basic emergency obstetric and newborn care
BU/RTC	Boston University/Right to Care Zambia
CBA	Controlled before-and-after
CEmONC	Comprehensive emergency obstetric and newborn care
GRZ	Government of the Republic of Zambia
HHS	Household survey
IDI	In-depth interview
IRB	Institutional review board
MHA	Maternity Homes Alliance
MMR	Maternal mortality ratio
MSD	Merck Sharp & Dohme
MWH	Maternity Waiting Home
PNC	Postnatal care
SDG	Sustainable Development Goals

For peer review only

ABSTRACT

Introduction: Maternity waiting homes (MWHs) aim to improve access to facility delivery in rural areas, especially among women living farthest from health facilities. However, there is limited rigorous evidence of their effectiveness. Using formative research, we developed a MWH intervention model with three components: infrastructure, management, and linkage to services. We present a protocol for a study that aims to measure the impact of the MWH model on facility delivery among women living ≥10km from their designated health facility in rural Zambia.

Methods and analysis: We are conducting a mixed-methods quasi-experimental impact evaluation of the MWH model using a controlled before-and-after design in 40 health facility clusters. Clusters were assigned to the intervention or control group using two methods: 20 clusters were randomly assigned using a matched-pair design; the other 20 were assigned without randomization due to local political constraints. Overall, 20 study clusters receive the MWH model intervention while 20 control clusters continue to implement the 'standard of care' for waiting mothers. We recruit a repeated cross-section of 2,400 randomly sampled recently-delivered women at baseline (2016) and endline (2018); all participants are administered a household survey and a 10% subsample also participates in an in-depth interview. The primary outcome is the probability of delivery at a health facility; secondary outcomes include utilization of MWHs and maternal and neonatal health outcomes.

Ethics: Ethical approvals were obtained from the Boston University Institutional Review Board (IRB), University of Michigan IRB (for de-identified data only), and the ERES Converge IRB in Zambia. Written informed consent is obtained prior to data collection.

Conclusion: To the best of our knowledge, Zambia is the first country to rigorously evaluate the impact of MWHs on women living most remotely. This study will generate key new evidence to inform decision making for MWH policy in Zambia and globally.

Trial Registration: ClinicalTrials.gov Identifier: NCT02620436

Keywords: maternity waiting home, maternal health, facility delivery, mixed methods, impact evaluation, Zambia

For peer review only

ARTICLE SUMMARY

Strengths and limitations of this study

This study has several strengths and limitations, including:

- To the best of our knowledge, this is the first large-scale impact evaluation of MWHs, employing a rigorous controlled before-and-after, quasi-experimental design and using mixed-methods.
- For generalizability, a representative sample of recently-delivered women living most remotely is selected using a multi-stage, random sampling strategy for both the quantitative household surveys and the qualitative in-depth interviews.
- Half of study clusters could not be randomly assigned to either the intervention or control group due to political constraints, resulting in quasi-experimental study design.
- Because remote women stand to benefit the most from the MWH model, eligibility is limited to those living at least 10km from the health facilities; findings will therefore not be able to assess impact of the intervention on women living nearer to facilities.
- In companion protocols, implementation fidelity of the core elements of the MWH model is assessed by each partner using harmonized tools.

For peer review only

INTRODUCTION

The Sustainable Development Goals (SDGs) include a target of reducing the global maternal mortality ratio (MMR) to less than 70 deaths per 100,000 live births by 2030.[1] Zambia's MMR is currently 398 deaths per 100,000 live births, well above the SDG target.[2,3] Skilled care at every birth, one of the two SDG indicators for MMR, is recommended. What remains unanswered is how to best facilitate access to intrapartum and postpartum care, particularly in rural and remote areas where distance and poor transportation severely restrict access to care. The Government of the Republic of Zambia (GRZ) is committed to improving maternal health and encourages facility-based delivery for all women,[4,5] though accessing facilities for birth is challenging for women living in remote areas.[6,7]

Maternity waiting homes (MWHs) are lodgings located near health facilities where mothers who are close to term can await delivery. MWHs are meant to provide pregnant women with the option of planning ahead and traveling to health facilities well before labor begins. MWHs may be a promising strategy to improve access to facilities for delivery, though there is limited rigorous evidence of their effectiveness on improving rates of facility delivery, particularly among women living remotely. While some evidence suggests MWHs are associated with higher rates of facility delivery and improved maternal health outcomes,[8,9] a Cochrane review found that there are no randomized or quasi-randomized trials assessing the effectiveness of MWHs in low-resource settings.[10] Rigorous evidence on the impact of MWHs on facility deliveries is needed.

This protocol describes a study being conducted by the Maternity Homes Alliance (MHA), a partnership between the GRZ, Boston University and Right to Care Zambia, formerly the Zambian Center for Applied Health Research and Development (BU/RTC), Africare and the University of Michigan (Africare/UM), and funded by Merck Sharp and Dohme (MSD) for Mothers, the Bill & Melinda Gates Foundation, and The ELMA Foundation. The MHA hypothesizes that MWHs can remove the distance barrier and increase access to facility-based delivery. In this study, we test the impact of MWHs on

For peer review only

facility delivery among women living at least 10km from health facilities in rural Zambia. To the best of our knowledge, this is the first study to rigorously evaluate the impact of MWHs on those living most remotely. This study will generate findings that contribute to the global knowledge of MWHs.

Intervention

Formative evaluations conducted previously by members of the study team in the current study setting showed that MWHs could be an acceptable and feasible option to improve access to facilities for delivery.[11–13] An MWH model, developed based on these findings, was designed to be responsive to community standards of acceptability including safety, comfort, and services offered in accordance with local input (Figure 1). The MWH model is community-owned and operated as per the request of government officials, but is operationally affiliated with the local health facility and is situated near the facility. Routine antenatal care (ANC) and other clinical services continue to be provided at the health facility, not in the MWH. The MWH model targets all pregnant women within 1-2 weeks of their estimated delivery date resident within the catchment area, prioritizing those women living fathest away (i.e. > 10 km from the health facility).

INSERT FIGURE 1

METHODS AND ANALYSIS

Evaluation questions

The primary research question is:

1. What is the impact of the MWH model on the probability of facility delivery among mothers living more than 10 km from the facility?

Secondary evaluation questions include:

For peer review only - http://bmjopen.bmj.com/site/about/guidelines.xhtml

1. Do awareness and perceptions of health facility-associated safe delivery and health facility delivery intention among pregnant women living in communities located more than 10 km from the health facility change over time in MWH model sites?
2. How do awareness and perceptions of MWHs by communities located more than 10 km from the health facility change over the period of this study?
3. What financial impact does the use of the MWH model have on the families of women who utilize it?
4. How does the perception of quality of care differ between MWH model sites and control sites?
5. What is the impact of the MWH model on maternal and neonatal health outcomes among those living more than 10 km from the facility?

Study setting

The intervention and comparison sites are located in the primarily rural Zambian districts of Choma, Kalomo and Pemba Districts of Southern Province; Nyimba and Lundazi Districts of Eastern Province; and Mansa and Chembe Districts of Luapula Province (Figure 2).

INSERT FIGURE 2

Choma district has a population of 247,860 and a population density of 34/km^2, with 68.7% of its population being rural. Kalomo district has a population of 258,570 and a population density of 17.2/km^2, with 91.8 percent of its population being rural.[14] Nyimba district has a population of 85,025 and a population density of 8.1/km^2, with 91 percent of its population being rural. Lundazi district has a population of 323,870 and a population density of 23/km^2, with 95.1 percent of its population being rural.[15] Mansa district has a population of 228,392 and a population density of 23.1/km^2, with 61.9 percent of its population being rural.[16]

For peer review only - http://bmjopen.bmj.com/site/about/guidelines.xhtml

Study design

This study employs a quasi-experimental controlled before-and-after (CBA) design with a total of 40 study clusters. Clusters are comprised of health facilities and their catchment households. Twenty intervention clusters receive the MWH model and 20 control clusters in the same districts continue to implement the 'standard of care' for waiting mothers. The current standard of care at facilities for waiting expectant mothers varies across Zambia: some have no designated space for a mother to wait; others have no MWH but provide a designated space for waiting mothers within the clinic; and a small number have an existing MWH-like structure but with highly variable quality.[9] Clusters were assigned to the intervention or control group using two methods: 20 clusters were randomly assigned (10 intervention and 10 control) using a matched-pair design, while the other 20 were assigned (10 intervention and 10 control) without randomization due to local political constraints (Table 1).

Table 1: Quasi-experimental study design to evaluate the impact of MWHs

**Randomized subsample (n=20 clusters)**	**Non-randomized subsample (n=20 clusters)**	**Non-randomized full sample (n=40 clusters)**
R O1 X O2	NR O1 X O2	NR O1 X O2
R O1 _ O2	NR O1 _ O2	NR O1 _ O2

X = Minimum Core Maternity Home (see above)
O = Observations at baseline (O1, in 2016) and endline (O2, in 2018) at intervention (X) and comparison (_) sites.
R = cluster randomized; NR = not randomized

Eligibility criteria of study sites

The MWH model is being implemented at 20 rural health facilities capable of managing basic emergency obstetric and neonatal complications (BEmONC). Facilities were eligible for inclusion in the study if they met at least one of two sets of conditions:

Eligibilty condition set 1:

i. Able to provide at least 5 of 7 BEmONC signal functions;

For peer review only

ii. ≤2 hours travel time to a comprehensive emergency obstetric and neonatal care (CEmONC) capable referral facility;

iii. Performs a minimum of 150 deliveries per year.

Eligiblity condition set 2:

i. Has at least one skilled birth attendant on staff;

ii. Routinely provide active management of third stage of labor;

iii. Has had no stock outs of oxytocin in the last 12 months;

iv. Has had no stock outs of magnesium sulfate in the last 12 months;

v. Located within ≤2 hours travel time to a CEmONC referral facility.

For the randomized subsample, 20 clusters were matched in pairs based on transfer time to CEmONC and clinic delivery volume. One cluster within each matched pair was then randomly selected to receive the intervention using the RAND function in Excel, yielding 10 intervention sites and 10 control sites. In the non-randomized subsample, 10 clusters were purposively selected to receive the intervention based on a consultative process with local stakeholders. Ten matched clusters were then identified from the full set of health facilities in the study districts based on transfer time to CEmONC and facility delivery volume.

Data sources

Population data are being collected from two main sources: household surveys (HHS) and in-depth interviews (IDIs). Baseline data collection occurred in early 2016 prior to the implementation of the MWH model in intervention clusters; endline data collection will occur in late 2018, after an 18 month intervention period. The HHS is administered to a sample of 2,400 recently delivered women (eligibility criteria described below) residing in intervention and control clusters. In the case of maternal

For peer review only

death, the household head or senior woman was interviewed as a proxy respondent. The HHS captures information on the domains and data fields seen in Table 2.

Table 2. Summary table of data fields collected from the household survey

**Household Panel:**	• Geo-coordinates of household/distance from nearest health facility<br>• Age and sex of household members<br>• Education level of household members<br>• Recent pregnancy/delivery of household members
**Individual Demographics and Household Characteristics**	• Number of pregnancies<br>• Outcome of pregnancies<br>• Number of living/deceased children<br>• Characteristics of living quarters (e.g., roof type, floor type, cooking fuel type)<br>• Access to and quality of water<br>• Household wealth indicators and assets
**Last Pregnancy**	• Antenatal services utilized<br>• HIV testing<br>○ Status<br>○ PMTCT*<br>• Perceived quality/satisfaction with Antenatal
**Last Delivery**	• Location of last delivery<br>○ Decision-maker in location of delivery<br>○ Mode of transportation<br>• Referral and bypassing<br>• Receipt of CEmONC services (C-section, blood transfusion)<br>• Perceived quality/satisfaction with delivery services<br>• Maternal and neonatal outcomes
**Use of Mother's Shelter**	• Knowledge of mother's shelter<br>• Nearest mother's shelter to home<br>• Use of mother's shelter before/after last delivery<br>○ Cost of using mother's shelter<br>○ Perceived quality of mother's shelter<br>○ Satisfaction with mother's shelter
**Cost of Delivery and Delivery Planning**	• Planned location for delivery<br>○ Adherence to planned location for delivery<br>• Savings for last delivery<br>• Cost of last delivery (broken down by expense)
**Postnatal Care (PNC)**	• Time to first maternal and newborn post-natal visit after delivery<br>• Perceived quality of post-natal services received<br>• Breastfeeding practices<br>• Supplementary feeding practices<br>• Newborn vaccination

For peer review only - http://bmjopen.bmj.com/site/about/guidelines.xhtml

	• PMTCT/ART[++] for newborn<br>• Interactions between the parent and the child<br>• Maternal depression assessment<br>• Health seeking behavior for child's last illness
**Health Care Knowledge and Beliefs**	• Use of contraceptive<br>• Primary barriers to accessing health care<br>○ Primary barriers to accessing skilled delivery services

[*]PMTCT = Prevention of mother-to-child transmission of HIV; [++]ART = Antiretroviral therapy

In-depth interviews are conducted among a subsample of 240 HHS respondents in order to gain a deeper understanding of community awareness, perceptions and experiences. Content includes perceptions of labor and delivery practices, barriers to accessing care, knowledge and awareness of MWHs, perceptions of the quality of maternity homes (guided by the MWH model), perceptions of MWH ownership, perceptions of quality of care at the facility, and expenses incurred for last delivery.

The population-based approach captures the experiences of those who utilized the facility in their catchment, other facilities, and those who did not access a facility for delivery, allowing us to more accurately estimate the impact of the MWH model intervention among women living farthest from the health facility.

Sampling strategy and sample size

We recruit a repeated cross-section of 2,400 households at each round for the survey (approximately 60 households per cluster): 1,200 from both intervention and control sites at both baseline (completed in 2016) and endline (planned for 2018), for a total study sample of 4,800 households (Table 3). After accounting for the clustered sampling design (ICC estimated at 0.04 based on previous work [17–19]), and assuming an alpha of .05, this sample will provide us with 80% power to detect a minimum 10 percentage point difference in the anticipated impact of the MWH intervention on the primary outcome of facility delivery, a programmatically meaningful difference. We recruited a sample of 240 women for the IDIs (randomly selecting 10% of the household sample) at baseline, and will recruit another 240 at endline.

For peer review only

For peer review only - http://bmjopen.bmj.com/site/about/guidelines.xhtml

Table 3: Total sample size for evaluation

Evaluation Activity	*Intervention Sites*	*Comparison Sites*	*Households per Site*	*X2 Observations (baseline & endline)*	*Total*
Household Survey	20	20	60	2	4800
*In-depth Interview**	*20*	*20*	*6*	*2*	480
TOTAL PARTICIPANTS FOR ALL EVALUATION ACTIVITIES:					**4800**
**Note that IDIs are a subset of the total household survey population selected for more in-depth information and are therefore NOT factored in as additional human subject participants in the total sample size for this study.*					

Participant recruitment

For the purposes of this evaluation, a household is defined as a group of people who regularly cook together. Inclusion criteria for the household survey are:

- Household with someone who has delivered a baby within the past 12 months
- Respondent must be age 15 or older. If age 15-17, a legal guardian must be available for consent.
- Proxy respondent (if woman deceased) must be over the age of 18
- Resident of the village identified for sampling (>10 km from the facility)

At baseline, conducted in 2016, we employed multi-stage random sampling procedures (Figure 3). We began the first stage of sampling by visiting every village within the catchment area of each study site, informing the local village leader of the purpose of the study and taking the GPS coordinates from the approximate geographical "center" of the village. We input these GPS coordinates into ArcGIS® Online (Esri, Redlands, CA) and used the line creation tool to draw the most direct route along the roads and paths visible on World Imagery basemap between each village center and their associated health facility. We then used this network of roads to determine the distance of each village to the health facility and developed a sampling frame of all villages within each catchment area located more than 10km from the health facility (rounding up from 9.5km). We then randomly selected a sample of 10

For peer review only - http://bmjopen.bmj.com/site/about/guidelines.xhtml

villages from each catchment area with probability proportional to population size. In the second stage of sampling, we worked with community volunteers and village leaders to list all households within the selected villages with a woman that had a delivery in the last year, randomly ordered them, visited each in that order and confirmed their eligibility for study participation. We continued down the list until the 6th eligible household in each village was identified. During the enrollment period, we selected additional villages and additional households within villages in order to reach our overall goal of 2,400 enrolled households.

INSERT FIGURE 3

The study team and community volunteers introduced the study to potential respondents and requested permission from the household head or most senior woman in the household to screen for eligibility. If household eligibility was confirmed, the study team proceeded with the informed voluntary consent process with the household head or senior woman. Once informed consent was obtained and documented from the household head or senior woman, the enumerator recorded the geo-location of the household and commenced the interview or scheduled a later appointment. The household head or senior woman responded to the first part of the survey for approximately 15 minutes, enumerating all of the people in the household in a table that captured demographics as well as recent deliveries and delivery outcomes.

Upon completion of the household demographics and enumeration, an eligible woman was selected to respond to the remainder of the survey. If more than one women in the household had delivered a baby in the past 12 months, the electronic data capture system randomly selected one eligible woman to respond to the remainder of the survey. The selected woman was then consented separately, enrolled in the study, and completed the HHS in a private space where she felt comfortable. Completion of the HHS took approximately 45-60 minutes.

For peer review only - http://bmjopen.bmj.com/site/about/guidelines.xhtml

Of the woman respondents, 10% were randomly selected to participate in a 30-minute IDI immediately following the survey. We will repeat the household-level sampling procedures at endline (O2 in 2018), selecting a new cross-sectional sample of households and women within the households. Therefore, the same households will not be followed over time. We will not re-geo-locate villages unless a new village has formed between baseline and endline.

Procedures

Data collection

At baseline, a local team of enumerators literate in the appropriate local language(s) and in English were trained in qualitative and quantitative research methods and human subjects' protection. Surveys were designed in SurveyCTO Collect software (Version 2.212; Dobility, Inc.) and captured electronically using encrypted tablets. The IDIs were digitally captured on audio recorders. Enumerators explained the tablet system to all respondents and explained the digital audio recorders to those selected for IDIs. These same methods will be followed at endline.

Checks were put in place to guarantee the quality of collected survey data. First, enumerators participated in an extensive 5-day training. Second, the enumerators were overseen by data collection team leads with greater experience in data collection fieldwork. Team leads were overseen by a field supervisor. Team leads and the field supervisors reviewed surveys for quality and completeness nightly. Third, field supervisors randomly selected a 5% subsample of households to be audited; the auditor revisited these households and repeated a subset of survey questions that were checked for reliability. Lastly, data were encrypted, uploaded and transferred nightly to the data analysis team where key consistency and quality tracking indicators were reviewed in real time. The same quality assurance methods will be followed at endline.

Data management

For peer review only - http://bmjopen.bmj.com/site/about/guidelines.xhtml

Survey data were captured on tablets and saved to the internal memory. Each evening, a data team supervisor reviewed the survey and encrypted it so survey data were no longer accessible on the tablet. The supervisor uploaded encrypted data nightly during the collection period to a secure server administered by SurveyCTO (Version 2.212; Dobility, Inc.). The evaluation team downloaded the encrypted data using the SurveyCTO Client software (Version 2.212; Dobility, Inc.), and decrypted the data using a decryption key generated by the research team.

The evaluation team oversaw data entry, management, and storage for qualitative data. All IDIs were translated into English and transcribed verbatim. Digital recorders and paper copies of written notes were kept in a locked cabinet until transcriptions were checked for quality and accuracy, at which point audio files were deleted and notes shredded. The electronic transcriptions do not contain identifying information, only a study ID number linked to their quantitative survey. A separate linking file for the quantitative and qualitative data is password protected and only accessible to the study team. All data management methods will be repeated at endline.

Data analysis

The primary independent variable of interest is assignment to the intervention. For the analysis, we will compare baseline characteristics between the intervention and control groups to assess balance. We collect data on potential confounders to increase precision, analyze heterogeneity, and, if necessary, control for any potential imbalance between the groups.

All quantitative analyses will be conducted in SAS v9.4 (SAS, Cary, NC). Our quantitative analytic plan is threefold, yielding descriptive, bivariate and multivariate statistics. First, we will describe the study sample, stratifying by intervention and control group and testing for differences between the groups. Second, we will estimate differences between the groups for primary and secondary outcomes. Categorical variables will be compared between the groups using a chi-squared test when cell sizes are sufficient or Fisher's exact test when the cell sizes are small; continuous variables will be compared

For peer review only

using t-tests if normally distributed or non-parametric Wilcoxon rank sum tests if the distribution is non-normal. Third, we will fit several regression models to estimate the impact of the intervention on the primary and secondary outcomes, adjusting for baseline values, assignment matching variables, and any imbalanced covariates.

The primary dependent variable is the probability of facility delivery for most recent birth, based on self-report by mothers. Secondary outcomes include:

- Use of MWHs for antenatal, delivery or postnatal services
- Delivery by cesaerean section
- Maternal death
- Neonatal death

All qualitative data will be analyzed in Nvivo 10 © software (QSR International Pty Ltd.). We will conduct a content analysis of the IDI transcripts. Coding themes have been identified *a priori*. Additional themes will be included as they emerge. We will triangulate findings with the quantitative data to identify consistencies, inconsistencies or additional themes to be explored. We will use the themes developed during the baseline analysis to analyze the endline data and identify any new themes as they emerge.

ETHICS AND DISSEMINATION

Ethics approval and consent to participate

Ethical review boards

Prior to participant enrollment, ethical approvals were obtained from the Boston University Institutional Review Board (IRB), University of Michigan IRB (for a de-identified dataset only), and the ERES Converge Research IRB, a private local ethics board in Zambia. We also obtained official approval to proceed with the study from the Zambia National Health Research Authority, which is responsible for oversight of all research conducted in the country. Adverse events, unanticipated problems and any protocol changes

For peer review only - http://bmjopen.bmj.com/site/about/guidelines.xhtml

will be reported to the IRBs and the Zambia National Health Research Authority per their guidelines, and all investigators will be informed.

Potential risks and protections

This study poses minimal risk to study participants and several steps were taken to minimize risk and burden. To reduce the risk of disclosure of personal or sensitive information enumerators are trained to stop participants from disclosing information that is too sensitive. Participation may cause some discomfort from answering certain questions, particularly if the maternal or neonatal health outcomes were adverse. Enumerators are trained to minimize any potential discomfort or harm to all participants during all study activities to the greatest extent possible. We minimize any waiting by participants by scheduling meetings during times convenient to participants and interviews are kept to as short of time as possible, though breaks are taken or follow-up meetings are scheduled as required. Participants received small tokens of appreciation valued around $1-2 (USD) in recognition of their time and opportunity costs.

Potential benefits

There are no direct individual benefits to participating in the study. The evaluation results will generate evidence on the impact of MWHs on facility delivery for those who live farthest away. Findings will provide insight for policy makers into how, if found to be effective, MWHs can be part of a broader strategy to improve maternal and neonatal health outcomes.

Respondent confidentiality

Throughout the study, we take care to ensure the confidentiality of data obtained from study participants. The HHSs and IDIs are carried out in participants' private homes or somewhere the respondent feels comfortable. We do not proceed with data collection until we can confirm that the location is acceptable and respondents agree that they feel comfortable discussing study topics.

For peer review only

For peer review only - http://bmjopen.bmj.com/site/about/guidelines.xhtml

The linking file with identifiable data and basic demographics is stored in a separate file within the tablet system. Upon completion of data collection, all files are stored on a secure server during data analysis and report writing. Only BU/RTC investigators have access to identifiable data. All analyses by study partners are conducted on de-identified datasets per IRB approvals. Analyses are presented in aggregate format in technical reports to stakeholders and in manuscripts submitted for publication in scientific journals. Under no circumstances do organizations or individuals have access to the participant's individual demographic information and potential identifying information (job title, age range, sex, and village). As explained above, the qualitative data are de-identified, with basic demographics only.

Informed consent

Prior to any data collection, we discuss the purpose of the study with local leaders so that the study activities are clearly understood. If a household is eligible, the study team proceeds with the informed voluntary consent process from the household head or the most senior woman in the household, introducing themselves, the purpose of the study, and explaining what we are asking of them in terms of participation, the risks and benefits, the right to withdraw without penalty at any time, that their information will be kept in a safe location, and that their answers will not be linked to their names. Participants are informed that the alternative is to not participate in the study. The study team slowly and clearly asks for consent to participate. If a selected household respondent declines participation, the next household on the randomly ordered list of eligible households is contacted. If a household head or senior woman consents to participating, the study team docuements written informed consent and proceeds with the interview. In addition to the household head or a senior woman, using the same process we also consent the woman selected from within the household to respond to the survey; in some cases, this may be the same person. A maximum of two individuals are consented per household.

For peer review only - http://bmjopen.bmj.com/site/about/guidelines.xhtml

We anticipate about 15% of the sample in each round to be between 15-17 years of age. In Zambia, 'emancipated minors' can enroll if they provide assent and their guardian or husband also provides consent. If a woman's husband is 18 or older, then he can provide informed consent on behalf of his wife; however, if he is also under 18 years old, then her legal guardian must provide consent. If under 18, the research team will allow the woman to first determine if she wishes to join the study (assent is provided) and then obtain consent by the guardian or husband. Thus, the individual's wishes are protected and she can determine if she wishes to be part of the study.

All informed consent or assent/consent is documented with a signature; in the event a respondent cannot write, a witness signs the informed consent. A participant retains a copy of the informed consent form. The informed consent and assent processes are always conducted in the language most preferred by the participant.

Costs and payments

For all activities, the participants volunteer only the time taken to complete this survey. There is no payment provided to participants for any portion of the study.

Dissemination of findings

The primary audience for this evaluation is the Government of Zambia, particularly the Ministry of Health, Ministry of Community Development, and the Ministry of Chiefs and Traditional Affairs, which will use the results to inform the development of maternal and child health strategies and policies in Zambia. We have disseminated the baseline findings to key stakeholders internal to Zambia and will disseminate the full study findings after endline. Many of the findings will likely be of broader interest throughout the region and globally where maternal mortality is high, resources are low, and access to facility-based delivery remains an issue. As such, results of this evaluation will be disseminated as widely as possible through open-access journals, websites, and international conferences.

For peer review only - http://bmjopen.bmj.com/site/about/guidelines.xhtml

Limitations

This study has several limitations. First, half of study clusters could not be randomly assigned to either the intervention or control group due to political constraints. We will analyze the full sample as a quasi-experimental CBA study. Additionally, we will estimate the impact in both the non-randomized and randomized subsamples and assess potential bias introduced by non-random assignment. Second, we limited household eligibility to those living at least 10 km from the health facilities and will not be able to assess impact on women living nearer to facilities. However, remote women are the primary target of the MWH model and stand to benefit the most from the intervention. To manage this limitation, in a separate protocol, we are collecting facility-based data to understand any changes in demographics among those utilizing facilities for delivery. Lastly, because there are two implementing partners, there is a risk that the MWH model will be implemented differently across the sites. To mitigate this risk, we have developed and agreed upon the precise elements of the MWH model based on both partners' formative research [11–13] and will be assessing implementation fidelity using harmonized tools.

CONCLUSION

Maternity Waiting Homes have the potential to improve access to facility delivery, particularly for women in rural areas living far from health facilities. Despite their widespread use in developing countries, there is currently little evidence of MWH effectiveness. Using community input, we developed a MWH model that responds to community-identified measures of acceptability, safety and comfort. To the best of our knowledge, this is the first large-scale impact evaluation of MWHs. Findings will be triangulated and explained by data from partner-specific process evaluations being implemented concurrently. Findings will generate evidence surrounding the effectiveness of MWHs on improving

For peer review only

For peer review only - http://bmjopen.bmj.com/site/about/guidelines.xhtml

facility deliveries for remote populations in Zambia and other countries with similar rural and highly dispersed populations.

REFERENCES

1 The United Nations. Goal 3 .:. Sustainable Development Knowledge Platform. https://sustainabledevelopment.un.org/sdg3 (accessed 2 Feb 2018).

2 Central Statistical Office (CSO) Zambia, [Ministry of Health (MOH) [Zambia], ICF International. Zambia Demographic and Health Survey 2013-14. Rockville, Maryland, USA: 2014.

3 UN General Assembly. Resolution adopted by the General Assembly on 25 September 2015 70/1. Transforming our world: the 2030 Agenda for Sustainable Development. 2030. http://www.un.org/en/development/desa/population/migration/generalassembly/docs/globalcompact/A_RES_70_1_E.pdf (accessed 17 Jan 2018).

4 Republic of Zambia Ministry of Health. National Health Strategic Plan 2011 - 2015. 2011;:119.

5 Republic of Zambia Ministry of Health. Roadmap for Accelerating Reduction of Maternal , Newborn and Child Mortality , 2013-2016. 2016;:2013–6.

6 Gabrysch S, Cousens S, Cox J, *et al.* The influence of distance and level of care on delivery place in rural Zambia: a study of linked national data in a geographic information system. *PLoS Med* 2011;**8**. doi:10.1371/journal.pmed.1000394

7 Gabrysch S, Campbell OM, Lawn J, *et al.* Still too far to walk: Literature review of the determinants of delivery service use. *BMC Pregnancy Childbirth* 2009;**9**:34. doi:10.1186/1471-2393-9-34

8 Lori JR, Wadsworth AC, Munro ML, *et al.* Promoting access: The use of maternity waiting homes to achieve safe motherhood. *Midwifery* 2013;**29**:1095–102. doi:10.1016/j.midw.2013.07.020

9 Henry EG, Semrau K, Hamer DH, *et al.* The influence of quality maternity waiting homes on utilization of facilities for delivery in rural Zambia. *Reprod Health* 2017;**14**:68. doi:10.1186/s12978-017-0328-z

10 van Lonkhuijzen L, Stekelenburg J, van Roosmalen J. Maternity waiting facilities for improving maternal and neonatal outcome in low-resource countries. *Cochrane database Syst Rev* 2012;**10**:CD006759. doi:10.1002/14651858.CD006759.pub3

11 Lori JR, Munro-Kramer ML, Mdluli EA, *et al.* Developing a community driven sustainable model of maternity waiting homes for rural Zambia. *Midwifery* 2016;**41**:89–95. doi:10.1016/j.midw.2016.08.005

12 Chibuye PS, Bazant ES, Wallon M, *et al.* Experiences with and expectations of maternity waiting homes in Luapula Province, Zambia: a mixed–methods, cross-sectional study with women, community groups and stakeholders. *BMC Pregnancy Childbirth* 2018;**18**:42. doi:10.1186/s12884-017-1649-1

13 Scott N, Haley C, Gille S. Improving equitable access to safe deliveries in Zambia: Findings from a formative evaluation. 2014.

For peer review only - http://bmjopen.bmj.com/site/about/guidelines.xhtml

14 Central Statistical Office (CSO) Zambia. 2010 Census of Population and Housing - Southern Province Analytical Report. Lusaka: 2014. http://web0.psa.gov.ph/statistics/census/population-and-housing

15 Central Statistical Office (CSO) Zambia. 2010 Census of Population and Housing - Eastern Province Analytical Report. Lusaka: 2014. https://www.zamstats.gov.zm/phocadownload/2010_Census/2010_Census_Analytical_Reports/Eastern Province Analytical Report - 2010 Census.pdf (accessed 19 Jan 2018).

16 Central Statistical Office (CSO) Zambia. Zambia 2010 Census of Population and Housing Population Summary Report. Lusaka: 2012. http://www.mcaz.gov.zm/wp-content/uploads/2014/10/2010-Census-of-Population-Summary-Report.pdf (accessed 19 Jan 2018).

17 Hamer DH, Herlihy JM, Musokotwane K, *et al.* Engagement of the Community, Traditional Leaders, and Public Health System in the Design and Implementation of a Large Community-Based, Cluster-Randomized Trial of Umbilical Cord Care in Zambia. *Am J Trop Med Hyg* 2015;**92**:666–72. doi:10.4269/ajtmh.14-0218

18 Semrau KEA, Herlihy J, Grogan C, *et al.* Effectiveness of 4% chlorhexidine umbilical cord care on neonatal mortality in Southern Province, Zambia (ZamCAT): a cluster-randomised controlled trial. *Lancet Glob Heal* 2016;**4**:e827–36. doi:10.1016/S2214-109X(16)30215-7

19 Pagel C, Prost A, Lewycka S, *et al.* Intracluster correlation coefficients and coefficients of variation for perinatal outcomes from five cluster-randomised controlled trials in low and middle-income countries: results and methodological implications. *Trials* 2011;**12**:151. doi:10.1186/1745-6215-12-151

AUTHORS STATEMENT

NS led the scientific design, implementation, and was primarily responsible for drafting this manuscript. JK contributed to the development of the protocol, led the development of the study sample, coordinated data collection, and contributed to revisions of the manuscript. TV and RB contributed to the revisions and science of the protocol and data collection instruments. RF contributed to the development of the protocol, implemented the electronic data capture system, and contributed to the revisions of this manuscript. TN, GB, CB, and JL provided feedback on the protocol and reviewed and edited the final manuscript. DH provided scientific support, technical input into the survey design and critically reviewed and edited the final manuscript. PR helped conceptualize the scientific design of the study, provided technical input into the survey design,

For peer review only

sampling approach, and critically reviewed and edited the final manuscript. All authors read and approved the final version of the manuscript.

FUNDING

This program was developed and is being implemented in collaboration with MSD for Mothers, MSD's 10-year, $500 million initiative to help create a world where no woman dies giving life. MSD for Mothers is an initiative of Merck & Co., Inc., Kenilworth, N.J., U.S.A. (MRK 1846-06500.COL). The development of this article was additionally supported in part by the Bill and Melinda Gates Foundation (OPP1130329) https://www.gatesfoundation.org/How-We-Work/Quick-Links/Grants-Database/Grants/2015/07/OPP1130329 and The ELMA foundation (ELMA-15-F0017) http://www.elmaphilanthropies.org/the-elma-foundation/. The funders had no role in study design, data collection and analysis, decision to publish, or preparation of the manuscript. The content is solely the responsibility of the authors and does not reflect positions or policies of MSD, the Bill & Melinda Gates Foundation or The ELMA Foundation.

CONFLICTS OF INTEREST STATEMENT

The authors declare that they have no competing interests.

FIGURE LEGEND

Figure 1. Minimum Maternity Waiting Home Model in the Maternity Home Alliance for Intervention Sites (n=20).

Figure 2. Map of the Maternity Home Alliance Intervention and Control Study Sites by Partner

Figure 3. Multi-stage Random Sampling Strategy for Baseline and Endline

For peer review only - http://bmjopen.bmj.com/site/about/guidelines.xhtml

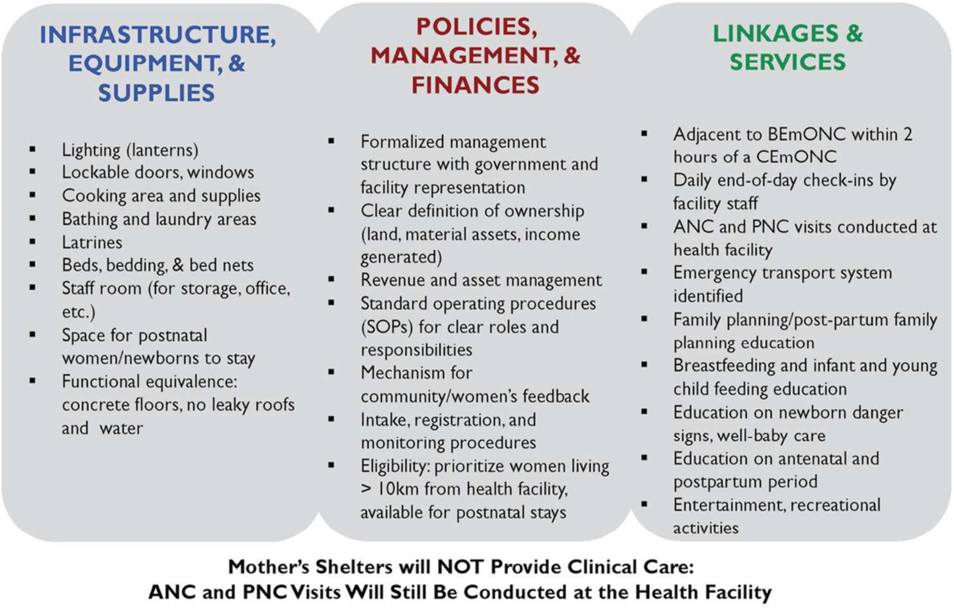

Figure 1. Minimum Maternity Waiting Home Model in the Maternity Home Alliance for Intervention Sites (n=20).

101x66mm (300 x 300 DPI)

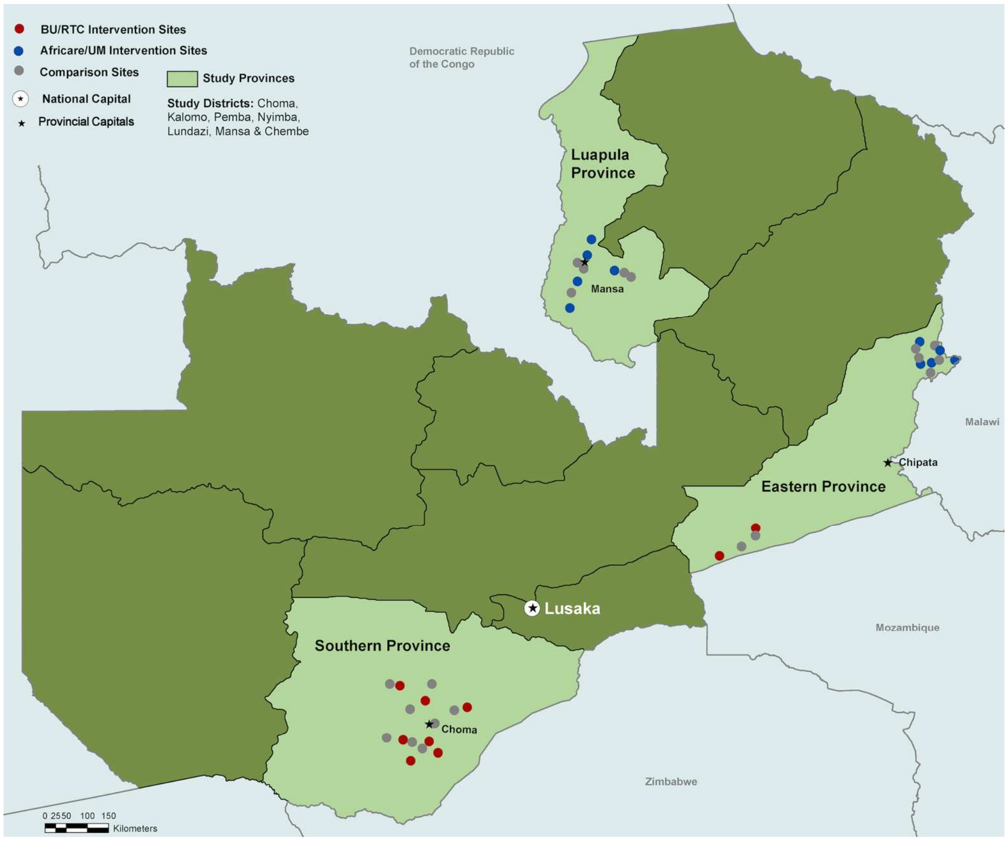

Figure 2. Map of the Maternity Home Alliance Intervention and Control Study Sites by Partner

173x144mm (300 x 300 DPI)

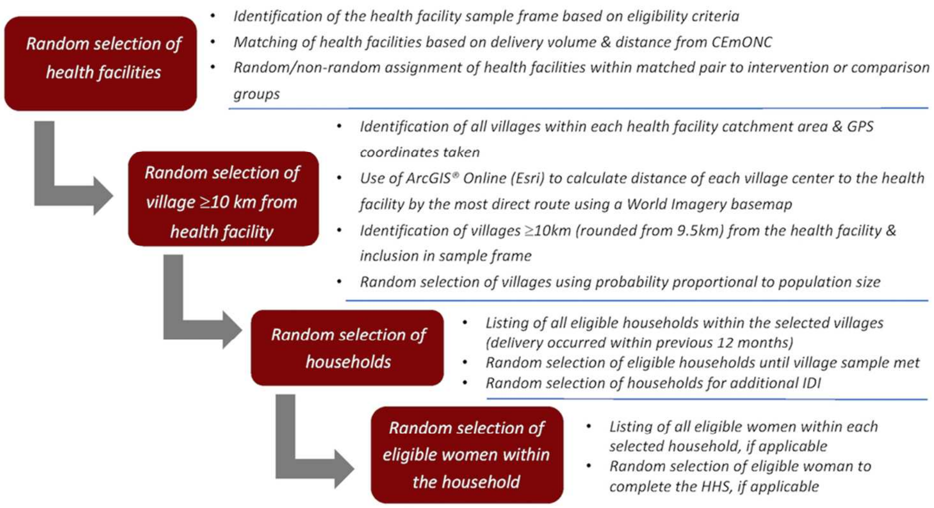

Figure 3. Multi-stage Random Sampling Strategy for Baseline and Endline

97x54mm (300 x 300 DPI)

SPIRIT

Standard Protocol Items: Recommendations for Interventional Trials

SPIRIT 2013 Checklist: Recommended items to address in a clinical trial protocol and related documents*

Section/item	Item No	Description	Addressed on page number
**Administrative information**			
Title	1	Descriptive title identifying the study design, population, interventions, and, if applicable, trial acronym	_____1________
Trial registration	2a	Trial identifier and registry name. If not yet registered, name of intended registry	_3,clinicaltrials.gov
	2b	All items from the World Health Organization Trial Registration Data Set	1-23 and on clinicaltrials.gov
Protocol version	3	Date and version identifier	______na_____
Funding	4	Sources and types of financial, material, and other support	______23_____
Roles and responsibilities	5a	Names, affiliations, and roles of protocol contributors	______1_______
	5b	Name and contact information for the trial sponsor	______23______
	5c	Role of study sponsor and funders, if any, in study design; collection, management, analysis, and interpretation of data; writing of the report; and the decision to submit the report for publication, including whether they will have ultimate authority over any of these activities	______23_______

For peer review only

For peer review only - http://bmjopen.bmj.com/site/about/guidelines.xhtml

	5d	Composition, roles, and responsibilities of the coordinating centre, steering committee, endpoint adjudication committee, data management team, and other individuals or groups overseeing the trial, if applicable (see Item 21a for data monitoring committee)	____na_________
**Introduction**			
Background and rationale	6a	Description of research question and justification for undertaking the trial, including summary of relevant studies (published and unpublished) examining benefits and harms for each intervention	_____5,6______
	6b	Explanation for choice of comparators	______8_______
Objectives	7	Specific objectives or hypotheses	_____5,6_______
Trial design	8	Description of trial design including type of trial (eg, parallel group, crossover, factorial, single group), allocation ratio, and framework (eg, superiority, equivalence, noninferiority, exploratory)	_____8________
**Methods: Participants, interventions, and outcomes**			
Study setting	9	Description of study settings (eg, community clinic, academic hospital) and list of countries where data will be collected. Reference to where list of study sites can be obtained	_____7________
Eligibility criteria	10	Inclusion and exclusion criteria for participants. If applicable, eligibility criteria for study centres and individuals who will perform the interventions (eg, surgeons, psychotherapists)	_____8,9,12____
Interventions	11a	Interventions for each group with sufficient detail to allow replication, including how and when they will be administered	______6_______
	11b	Criteria for discontinuing or modifying allocated interventions for a given trial participant (eg, drug dose change in response to harms, participant request, or improving/worsening disease)	______na_______
	11c	Strategies to improve adherence to intervention protocols, and any procedures for monitoring adherence (eg, drug tablet return, laboratory tests)	_____na________
	11d	Relevant concomitant care and interventions that are permitted or prohibited during the trial	______na_______

For peer review only - http://bmjopen.bmj.com/site/about/guidelines.xhtml

Outcomes	12	Primary, secondary, and other outcomes, including the specific measurement variable (eg, systolic blood pressure), analysis metric (eg, change from baseline, final value, time to event), method of aggregation (eg, median, proportion), and time point for each outcome. Explanation of the clinical relevance of chosen efficacy and harm outcomes is strongly recommended	____6,7,16______
Participant timeline	13	Time schedule of enrolment, interventions (including any run-ins and washouts), assessments, and visits for participants. A schematic diagram is highly recommended (see Figure)	_____11_______
Sample size	14	Estimated number of participants needed to achieve study objectives and how it was determined, including clinical and statistical assumptions supporting any sample size calculations	_____11________
Recruitment	15	Strategies for achieving adequate participant enrolment to reach target sample size	_____13________
**Methods: Assignment of interventions (for controlled trials)**			
Allocation:			
Sequence generation	16a	Method of generating the allocation sequence (eg, computer-generated random numbers), and list of any factors for stratification. To reduce predictability of a random sequence, details of any planned restriction (eg, blocking) should be provided in a separate document that is unavailable to those who enrol participants or assign interventions	____9, 12, 13, __
Allocation concealment mechanism	16b	Mechanism of implementing the allocation sequence (eg, central telephone; sequentially numbered, opaque, sealed envelopes), describing any steps to conceal the sequence until interventions are assigned	______na_______
Implementation	16c	Who will generate the allocation sequence, who will enrol participants, and who will assign participants to interventions	_____na________
Blinding (masking)	17a	Who will be blinded after assignment to interventions (eg, trial participants, care providers, outcome assessors, data analysts), and how	______na_______
	17b	If blinded, circumstances under which unblinding is permissible, and procedure for revealing a participant's allocated intervention during the trial	______na_______
**Methods: Data collection, management, and analysis**			

Data collection methods	18a	Plans for assessment and collection of outcome, baseline, and other trial data, including any related processes to promote data quality (eg, duplicate measurements, training of assessors) and a description of study instruments (eg, questionnaires, laboratory tests) along with their reliability and validity, if known. Reference to where data collection forms can be found, if not in the protocol	____6-14_______
	18b	Plans to promote participant retention and complete follow-up, including list of any outcome data to be collected for participants who discontinue or deviate from intervention protocols	_____na________
Data management	19	Plans for data entry, coding, security, and storage, including any related processes to promote data quality (eg, double data entry; range checks for data values). Reference to where details of data management procedures can be found, if not in the protocol	____14, 15______
Statistical methods	20a	Statistical methods for analysing primary and secondary outcomes. Reference to where other details of the statistical analysis plan can be found, if not in the protocol	_____15, 16_____
	20b	Methods for any additional analyses (eg, subgroup and adjusted analyses)	______16_______
	20c	Definition of analysis population relating to protocol non-adherence (eg, as randomised analysis), and any statistical methods to handle missing data (eg, multiple imputation)	______na_______

Methods: Monitoring

Data monitoring	21a	Composition of data monitoring committee (DMC); summary of its role and reporting structure; statement of whether it is independent from the sponsor and competing interests; and reference to where further details about its charter can be found, if not in the protocol. Alternatively, an explanation of why a DMC is not needed	_____na________
	21b	Description of any interim analyses and stopping guidelines, including who will have access to these interim results and make the final decision to terminate the trial	______na_______
Harms	22	Plans for collecting, assessing, reporting, and managing solicited and spontaneously reported adverse events and other unintended effects of trial interventions or trial conduct	______16, 17____
Auditing	23	Frequency and procedures for auditing trial conduct, if any, and whether the process will be independent from investigators and the sponsor	______na_______

Ethics and dissemination

For peer review only - http://bmjopen.bmj.com/site/about/guidelines.xhtml

Research ethics approval	24	Plans for seeking research ethics committee/institutional review board (REC/IRB) approval	______16_______
Protocol amendments	25	Plans for communicating important protocol modifications (eg, changes to eligibility criteria, outcomes, analyses) to relevant parties (eg, investigators, REC/IRBs, trial participants, trial registries, journals, regulators)	______16, 17____
Consent or assent	26a	Who will obtain informed consent or assent from potential trial participants or authorised surrogates, and how (see Item 32)	____18, 19______
	26b	Additional consent provisions for collection and use of participant data and biological specimens in ancillary studies, if applicable	_____na________
Confidentiality	27	How personal information about potential and enrolled participants will be collected, shared, and maintained in order to protect confidentiality before, during, and after the trial	____17, 18______
Declaration of interests	28	Financial and other competing interests for principal investigators for the overall trial and each study site	________23______
Access to data	29	Statement of who will have access to the final trial dataset, and disclosure of contractual agreements that limit such access for investigators	_____18________
Ancillary and post-trial care	30	Provisions, if any, for ancillary and post-trial care, and for compensation to those who suffer harm from trial participation	_____na________
Dissemination policy	31a	Plans for investigators and sponsor to communicate trial results to participants, healthcare professionals, the public, and other relevant groups (eg, via publication, reporting in results databases, or other data sharing arrangements), including any publication restrictions	______19_______
	31b	Authorship eligibility guidelines and any intended use of professional writers	_____22, 23_____
	31c	Plans, if any, for granting public access to the full protocol, participant-level dataset, and statistical code	______na_______
**Appendices**			
Informed consent materials	32	Model consent form and other related documentation given to participants and authorised surrogates	_Can be provided upon request__

5

For peer review only

Biological specimens	33	Plans for collection, laboratory evaluation, and storage of biological specimens for genetic or molecular analysis in the current trial and for future use in ancillary studies, if applicable	_____na________

*It is strongly recommended that this checklist be read in conjunction with the SPIRIT 2013 Explanation & Elaboration for important clarification on the items. Amendments to the protocol should be tracked and dated. The SPIRIT checklist is copyrighted by the SPIRIT Group under the Creative Commons "Attribution-NonCommercial-NoDerivs 3.0 Unported" license.

For peer review only

BMJ Open

Impact of maternity waiting homes on facility delivery among remote households in Zambia: protocol for a quasi-experimental, mixed-methods study

Journal:	*BMJ Open*
Manuscript ID	bmjopen-2018-022224.R1
Article Type:	Protocol
Date Submitted by the Author:	31-May-2018

Complete List of Authors:	Scott, Nancy; Boston University School of Public Health, Global Health<br>Kaiser, Jeanette; Boston University School of Public Health, Global Health<br>Vian, Taryn; Boston University School of Public Health, Global Health<br>Bonawitz, Rachael; Boston University School of Public Health, Global Health; Boston University School of Medicine, Pediatrics<br>Fong, Rachel; Boston University School of Public Health, Global Health<br>Ngoma, Thandiwe; Right to Care Zambia<br>Biemba, Godfrey; National Health Research Authority, Pediatric Centre of Excellence<br>Boyd, Carol; University of Michigan School of Nursing, Health Behavior & Biological Sciences<br>Lori, Jody; University of Michigan School of Nursing, Center for Global Affairs & PAHO/WHO Collaborating Center<br>Hamer, D.; Boston University,<br>Rockers, Peter; Boston University, Department of Global Health

**Primary Subject Heading**:	Obstetrics and gynaecology
Secondary Subject Heading:	Research methods, Global health, Qualitative research
Keywords:	maternity waiting home, maternal health, skilled birth attendance, mixed methods, impact evaluation, Zambia

SCHOLARONE™ Manuscripts

Title: *Impact of maternity waiting homes on facility delivery among remote households in Zambia: protocol for a quasi-experimental, mixed-methods study*

Authors:

Name	Institutional Address	Email Address
Nancy Scott	Department of Global Health, Boston University School of Public Health, Boston, USA<br>801 Massachusetts Ave, Boston, MA 02118	nscott@bu.edu (corresponding)<br>Tel: +1-617-414-1278
Jeanette Kaiser	Department of Global Health, Boston University School of Public Health<br>Boston, MA USA	jlkaiser@bu.edu
Taryn Vian	Department of Global Health, Boston University School of Public Health<br>Boston, MA USA	tvian@bu.edu
Rachael Bonawitz	Department of Global Health, Boston University School of Public Health<br>Boston, MA USA<br>Department of Pediatrics, Boston University School of Medicine<br>Boston, MA USA	rebonawi@bu.edu
Rachel Fong	Department of Global Health, Boston University School of Public Health<br>Boston, MA USA	rmfong@bu.edu
Thandiwe Ngoma	Right to Care-Zambia<br>Lusaka, Zambia	ngoma.thandiwe@gmail.com
Godfrey Biemba	National Health Research Authority, Pediatric Centre of Excellence<br>Lusaka, Zambia	gbiemba@gmail.com
Carol J. Boyd	Department of Health Behavior & Biological Sciences<br>School of Nursing, University of Michigan<br>Ann Arbor, MI USA	caroboyd@med.umich.edu
Jody R. Lori	Center for Global Affairs &<br>PAHO/WHO Collaborating Center<br>School of Nursing, University of Michigan<br>Ann Arbor, MI USA	jrlori@umich.edu
Davidson H. Hamer	Department of Global Health, Boston University School of Public Health<br>Boston, MA USA<br><br>Section of Infectious Diseases, Department of Medicine, Boston Medical Center<br>Boston, MA USA	dhamer@bu.edu
Peter C. Rockers	Department of Global Health, Boston University School of Public Health<br>Boston, MA USA	prockers@bu.edu

Word count: 4,200 (excluding title page, abstract, references, figures and tables)

For peer review only

List of Acronyms

Africare/UM	Africare/University of Michigan
ANC	Antenatal care
BEmONC	Basic emergency obstetric and newborn care
BU/RTC	Boston University/Right to Care Zambia
CBA	Controlled before-and-after
CEmONC	Comprehensive emergency obstetric and newborn care
GRZ	Government of the Republic of Zambia
HHS	Household survey
IDI	In-depth interview
IRB	Institutional review board
MHA	Maternity Homes Alliance
MMR	Maternal mortality ratio
MSD	Merck Sharp & Dohme
MWH	Maternity Waiting Home
PNC	Postnatal care
SDG	Sustainable Development Goals

For peer review only

ABSTRACT

Introduction: Maternity waiting homes (MWHs) aim to improve access to facility delivery in rural areas. However, there is limited rigorous evidence of their effectiveness. Using formative research, we developed a MWH intervention model with three components: infrastructure, management, and linkage to services. This protocol describes a study to measure the impact of the MWH model on facility delivery among women living farthest (≥10km) from their designated health facility in rural Zambia. This study will generate key new evidence to inform decision making for MWH policy in Zambia and globally.

Methods and analysis: We are conducting a mixed-methods quasi-experimental impact evaluation of the MWH model using a controlled before-and-after design in 40 health facility clusters. Clusters were assigned to the intervention or control group using two methods: 20 clusters were randomly assigned using a matched-pair design; the other 20 were assigned without randomization due to local political constraints. Overall, 20 study clusters receive the MWH model intervention while 20 control clusters continue to implement the 'standard of care' for waiting mothers. We recruit a repeated cross-section of 2,400 randomly sampled recently-delivered women at baseline (2016) and endline (2018); all participants are administered a household survey and a 10% subsample also participates in an in-depth interview. We will calculate descriptive statistics and adjusted odds ratios; qualitative data will be analyzed using content analysis. The primary outcome is the probability of delivery at a health facility; secondary outcomes include utilization of MWHs and maternal and neonatal health outcomes.

Ethics and dissemination: Ethical approvals were obtained from the Boston University Institutional Review Board (IRB), University of Michigan IRB (de-identified data only), and the ERES Converge IRB in Zambia. Written informed consent is obtained prior to data collection. Results will be disseminated to key stakeholders in Zambia, then through open-access journals, websites, and international conferences.

Trial Registration: ClinicalTrials.gov Identifier: NCT02620436

Keywords: maternity waiting home, maternal health, facility delivery, mixed methods, impact evaluation, Zambia

For peer review only

ARTICLE SUMMARY

Strengths and limitations of this study

This study has several strengths and limitations, including:

- To the best of our knowledge, this is the first large-scale impact evaluation of MWHs, employing a rigorous controlled before-and-after, quasi-experimental design and using mixed-methods.
- For generalizability, a representative sample of recently-delivered women living most remotely is selected using a multi-stage, random sampling strategy for both the quantitative household surveys and the qualitative in-depth interviews.
- Half of study clusters could not be randomly assigned to either the intervention or control group due to political constraints, resulting in quasi-experimental study design.
- Because remote women stand to benefit the most from the MWH model, eligibility is limited to those living at least 10km from the health facilities; findings will therefore not be able to assess impact of the intervention on women living nearer to facilities.
- In companion protocols, implementation fidelity of the core elements of the MWH model is assessed by each partner using harmonized tools.

For peer review only - http://bmjopen.bmj.com/site/about/guidelines.xhtml

INTRODUCTION

The Sustainable Development Goals (SDGs) include a target of reducing the global maternal mortality ratio (MMR) to less than 70 deaths per 100,000 live births by 2030.[1] Zambia's MMR is currently 398 deaths per 100,000 live births, well above the SDG target.[2,3] Skilled care at every birth, one of the two SDG indicators for MMR, is recommended. What remains unanswered is how to best facilitate access to intrapartum and postpartum care, particularly in rural and remote areas where distance and poor transportation severely restrict access to care. The Government of the Republic of Zambia (GRZ) is committed to improving maternal health and encourages facility-based delivery for all women,[4,5] though accessing facilities for birth is challenging for women living in remote areas.[6–9]

Maternity waiting homes (MWHs) are lodgings located near health facilities where mothers who are close to term can await delivery. These homes are meant to provide pregnant women with the option of planning ahead and traveling to health facilities well before labor begins. MWHs may be a promising strategy to improve access to facilities for delivery, the evidence is mixed. While some evidence suggests they are associated with higher rates of facility delivery and improved maternal health outcomes,[10–20] a Cochrane review found that there are no randomized or quasi-randomized trials assessing the effectiveness of MWHs in low-resource settings.[21] Additionally, it is unclear if MWHs can increase access to facility delivery among women living most remotely.[19,22] Rigorous evidence on the impact of MWHs on facility deliveries is needed.

This protocol describes a study being conducted by the Maternity Homes Alliance (MHA), a partnership between the GRZ, Boston University and Right to Care Zambia, formerly the Zambian Center for Applied Health Research and Development (BU/RTC), Africare and the University of Michigan (Africare/UM), and funded by Merck Sharp and Dohme (MSD) for Mothers, the Bill & Melinda Gates Foundation, and The ELMA Foundation. The MHA hypothesizes that MWHs can remove the distance

For peer review only - http://bmjopen.bmj.com/site/about/guidelines.xhtml

barrier and increase access to facility-based delivery. In this study, we test the impact of MWHs on facility delivery among women living at least 10km from health facilities in rural Zambia.

MWHs have the potential to improve access to facility delivery, particularly for women in rural areas living far from health facilities. Despite their widespread use in developing countries, there is currently little evidence of MWH effectiveness. Using community input, we developed a MWH model and are evaluating it for impact. To the best of our knowledge, this is the first large-scale impact evaluation of MWHs. Findings will generate evidence surrounding the effectiveness of MWHs on improving facility deliveries for remote populations in Zambia and other countries with similar rural and highly dispersed populations.

Intervention

While the government of Zambia supports the use of MWHs as a strategic method to increase access to skilled birth attendance [5,23] and MWHs have existed in Zambia for decades, there is no specific policy or plan for the scale-up of MWHs and their general quality remains low.[13,24–26] MWHs have been largely constructed through community initiatives or international donors, with limited support for their long-term maintenance.[24–26] Formative evaluations conducted previously by members of the study team in the current study setting showed that MWHs could be an acceptable and feasible option to improve access to facilities for delivery.[24–26] Informed by these findings, the core MWH model was designed to be responsive to community expectations, community-defined standards of acceptability and their perceptions of quality including safety, comfort, management and services offered (Figure 1). In direct response to the formative data, the model includes the following:

- **Infrastructure, Supplies and Equipment:** The core MWH model has concrete walls and floors, roofs that do not leak, latrines, a private bathing space, water within a reasonable distance, a covered cooking space, and storage space. For safety, the core MWH model has lockable doors,

For peer review only

windows, cupboards, and lighting. Amenities include beds, mattresses, bedding, mosquito nets, and cooking utensils.

- **Policies, Management and Finances:** The core MWH model is community-owned and operated, as requested by the Ministry of Health. The policies, management, and financial structures are adaptable to site-specific needs and preferences, though all have a formalized governance and management structure with community, government and health facility representation. Each also has a management unit responsible for daily operations including registering and orienting women, record keeping and maintenance.
- **Linkages & Services:** Each core MWH model is situated close to the health facility to ensure timely access to clinical care when a woman's labor begins. A health facility staff provides daily check-ins with waiting women, though clinical care visits continue to be conducted at the health facility, not in the MWH. Women staying at the MWH have the opportunity to participate in maternal and child education courses offered by the health facility staff or community health workers.

INSERT FIGURE 1

The core MWH model is promoted in the community through several mechanisms. First, health facility staff promote the MWH at all ANC visits. Over 95% of women attend at least the first ANC visit , so most women are exposed at the health facility.[2] Second, Safe Motherhood Action Group members promote the use of MWHs during their routine outreach activities. Lastly, the traditional leadership (chiefs and headmen) actively promotes the use of MWHs at their community meetings. The MWH model targets all pregnant women within 1-2 weeks of their estimated delivery date resident within the

For peer review only

catchment area, prioritizing those women living farthest away (i.e. > 10 km from the health facility). The 20 MWHs opened in phases between mid-2016 and mid-2017.

METHODS

Evaluation questions

The primary research question is:

1. What is the impact of the MWH model on the probability of facility delivery among mothers living more than 10 km from the health facility?

Secondary questions include:

1. Do awareness and perceptions of health facility-associated safe delivery and health facility delivery intention among pregnant women living in communities located more than 10 km from the health facility change over time in MWH model sites?
2. How do awareness and perceptions of MWHs by communities located more than 10 km from the health facility change over the period of this study?
3. What financial impact does the use of the MWH model have on the families of women who utilize it?
4. How does the perception of quality and acceptability differ between MWH model sites and comparison sites?
5. What is the impact of the MWH model on maternal and neonatal health outcomes among those living more than 10 km from the facility?

Study setting

This study began in March 2016 and will be completed in December 2018. The intervention and comparison sites are located in the primarily rural Zambian districts of Choma, Kalomo and Pemba

For peer review only

Districts of Southern Province; Nyimba and Lundazi Districts of Eastern Province; and Mansa and Chembe Districts of Luapula Province (Figure 2).

INSERT FIGURE 2

Choma district has a population of 247,860 and a population density of 34/km^2, with 68.7% of its population being rural. Kalomo district has a population of 258,570 and a population density of 17.2/km^2, with 91.8 percent of its population being rural.[27] Nyimba district has a population of 85,025 and a population density of 8.1/km^2, with 91 percent of its population being rural. Lundazi district has a population of 323,870 and a population density of 23/km^2, with 95.1 percent of its population being rural.[28] Mansa district has a population of 228,392 and a population density of 23.1/km^2, with 61.9 percent of its population being rural.[29]

Study design

This study employs a quasi-experimental controlled before-and-after (CBA) design with a total of 40 study clusters, 20 intervention and 20 control clusters.. Clusters consist of health facilities and their catchment households. Intervention clusters are receiving the MWH model, inclusive of newly constructed homes with the elements from the three domains: (1) infrastructure, equipment and supplies; 2) policies, management and finances; and 3) linkages and services detailed in the intervention section of the protocol. Control clusters are implementing the 'standard of care' for waiting mothers in Zambia. Because no national policy exists, the standard of care is facility-driven and varies widely. Some standard-of-care facilities have no designated space for a mother to wait; others have no MWH but provide a designated space for waiting mothers within the clinic; and a small number have an existing MWH-like structure but with highly variable quality.[13]

For peer review only - http://bmjopen.bmj.com/site/about/guidelines.xhtml

Eligibility criteria of study clusters

Because the intervention aims to generate demand for health facility delivery, it it criticle that facilities are capable of managing basic emergency obstetric and neonatal complications (BEmONC). Because of inconsistencies in available secondary data sources across the different districts, we established supplemental criteria that could be drawn from the available sources.[30,31] Clusters were eligible for inclusion in the study if the health facility was located ≤2 hours driving time to a comprehensive emergency obstetric and neonatal care (CEmONC) capable referral facility, performed a minimum of 150 deliveries per year AND met at least one of two sets of conditions below:

Eligibility condition set 1:

i. Facility is able to provide at least 5 of 7 BEmONC signal functions based on 2015 data;

Eligibility condition set 2:

i. Facility has at least one skilled birth attendant on staff;
ii. Facility routinely provide active management of third stage of labor;
iii. Facilty has had no stock outs of oxytocin in the last 12 months;
iv. Facility has had no stock outs of magnesium sulfate in the last 12 months;

Selection and assignment of study clusters to study arm

There are a total of 40 clusters (20 intervention, 20 comparison) in this study (Table 1). Each implementing partner used different methods to select and assign clusters to study arms. One partner had a total of 36 eligible health facilities that were located ≤2 hours driving time to a referral facility and performed a minimum of 150 deliveries. Of those, 22 (61%) met one of the two elgibility conditions. This partner selected the 20 farthest away from referral, created 10 pairs matched on annual delivery

For peer review only - http://bmjopen.bmj.com/site/about/guidelines.xhtml

volume and distance, then randomized matched pairs to intervention or control, using the RAND function in Microsoft Excel®.

Table 1: Quasi-experimental study design to evaluate the impact of MWHs

**Randomized subsample (n=20 clusters)**	**Non-randomized subsample (n=20 clusters)**	**Non-randomized full sample (n=40 clusters)**
R O1 X O2	NR O1 X O2	NR O1 X O2
R O1 _ O2	NR O1 _ O2	NR O1 _ O2

X = Minimum Core Maternity Home (see above)
O = Observations at baseline (O1, in 2016) and endline (O2, in 2018) at intervention (X) and comparison (_) sites.
R = cluster randomized; NR = not randomized

The other partner had a total of 29 eligible health facilities that were located ≤2 hours driving time to a referral facility and performed a minimum of 150 deliveries. Of those, 22 (76%) met one of the two sets of eligibility conditions. This partner was unable to randomly allocate sites to a study arm due to local political constraints, as the Ministry of Health feared community fatigue due to the large number of organizations implementing projects and conducting research. As such, the partner worked with the Ministry of Health to identify 10 intervention sites. From the remaining eligible, they excluded those with an existing functional MWH, then selected comparison sites matched on annual delivery volume and distance to a referral hospital.

Data sources

Population data are being collected from two main sources: household surveys (HHS) and in-depth interviews (IDIs). Baseline data collection occurred in early 2016 prior to the implementation of the MWH model in intervention clusters; endline data collection will occur in late 2018, after an 18 month intervention period. The HHS is administered to a sample of 2,400 recently delivered women (eligibility criteria described below) residing in intervention and control clusters. In the case of maternal death, the household head or senior woman was interviewed as a proxy participant.

For peer review only - http://bmjopen.bmj.com/site/about/guidelines.xhtml

The HHS captures information on the domains and data fields seen in Table 2. The HHS was pretested among a sample of 50 participants representing all the major local languages. At baseline, only small adjustments were made in response to the pre-test, primarily changing formal translations into the vernacular.

Table 2. Summary table of data fields collected from the household survey

**Household Panel:**	• Geo-coordinates of household/distance from nearest health facility<br>• Age and sex of household members<br>• Education level of household members<br>• Recent pregnancy/delivery of household members
**Individual Demographics and Household Characteristics**	• Number of pregnancies<br>• Outcome of pregnancies<br>• Number of living/deceased children<br>• Characteristics of living quarters (e.g., roof type, floor type, cooking fuel type)<br>• Access to and quality of water<br>• Household wealth indicators and assets
**Last Pregnancy**	• Antenatal care services utilized<br>• HIV testing<br>○ Status<br>○ PMTCT*<br>• Perceived satisfaction with antenatal care
**Last Delivery**	• Location of last delivery<br>○ Decision-maker in location of delivery<br>○ Mode of transportation<br>• Referral and bypassing<br>• Receipt of CEmONC services (C-section, blood transfusion, IV antibitotics)<br>• Perceived quality/satisfaction with delivery services<br>• Maternal and neonatal vital status
**Use of MWH**	• Knowledge of MWH<br>• Source of knowledge of MWH<br>• Nearest MWH to home<br>• Use of MWH before/after last delivery<br>○ Cost of using mother's shelter<br>○ Perceived quality of mother's shelter (safety, comfort, management and services)<br>○ Satisfaction with mother's shelter<br>• Prior use of MWH<br>• Intented future use of MWH
**Cost of Delivery and Delivery**	• Planned or intended location for delivery

For peer review only - http://bmjopen.bmj.com/site/about/guidelines.xhtml

**Planning**	○ Adherence to planned or intended location for delivery<br>• Barriers to birth plan adherence<br>• Savings for last delivery<br>• Cost of last delivery (broken down by expense)
**Postnatal Care (PNC) and infant health**	• Time to first maternal and newborn post-natal visit after delivery<br>• Perceived quality of post-natal services received<br>• Breastfeeding practices<br>• Supplementary feeding practices<br>• Newborn vaccination status<br>• PMTCT/ART[++] for newborn<br>• Interactions between the parent and the child<br>• Maternal depression assessment<br>• Health seeking behavior for child's last illness
**Health Care Knowledge and Beliefs**	• Use of contraceptives for family planning<br>• Primary barriers to accessing health care<br>○ Primary barriers to accessing skilled delivery services

[*]PMTCT = Prevention of mother-to-child transmission of HIV; [++]ART = Antiretroviral therapy

In-depth interviews are conducted among a subsample of 240 HHS participants in order to gain a deeper understanding of community awareness, perceptions and experiences. Because the seven districts are spread out and culturally different, we wanted to ensure we reached saturation or predictability in each district to better explore context with the qualitative data.[32] Consequently, we planned to conduct a large number of in-depth interviews to make sure there was sufficient coverage of different populations to provide insight into the quantitive survey findings. IDI content builds upon themes captured in the HHS and includes perceptions of labor and delivery practices, barriers to accessing care, knowledge and awareness of MWHs, sources of knowledge of MWH, perceptions of the quality of maternity homes (safety, comfort, management and services), perceptions of MWH ownership, perceptions of health facility, and expenses incurred for last delivery.

The population-based approach captures the experiences of those who utilized the facility in their catchment, other facilities, and those who did not access a facility for delivery, allowing us to more accurately estimate the impact of the MWH model intervention among women living farthest from the health facility in an intention-to-treat analysis.

For peer review only

Sampling strategy and sample size

To estimate the impact of the MWH model based on an intention-to-treat analysis, we aim to select a representative sample of women in our sample frame who delivered a baby in the past 12 months, irrespective of her place of delivery or her use of a MWH. With this strategy, we will also be able to explore the relationship between use of the MWH and location of delivery. As such, we are recruiting a repeated cross-section of 2,400 households at each round for the survey (approximately 60 households per cluster): 1,200 from both intervention and control sites at both baseline (completed in 2016) and endline (planned for 2018), for a total study sample of 4,800 households (Table 3).

Table 3: Total sample size for evaluation

Evaluation Activity	*Intervention Sites*	*Comparison Sites*	*Households per Site*	*X2 Observations (baseline & endline)*	*Total*
Household Survey	20	20	60	2	4800
*In-depth Interview**	*20*	*20*	*6*	*2*	480
			TOTAL PARTICIPANTS FOR ALL EVALUATION ACTIVITIES:		**4800**

**Note that IDIs are a subset of the total household survey population selected for more in-depth information and are therefore NOT factored in as additional human subject participants in the total sample size for this study.*

After accounting for the clustered sampling design (ICC estimated at 0.04 based on previous work [33–35]), and assuming an alpha of .05, this sample will provide us with 80% power to detect a minimum 10 percentage point difference in the anticipated impact of the MWH intervention on the primary outcome of facility delivery, a programmatically meaningful difference. We recruited a sample of 240 women for the IDIs (randomly selecting 10% of the household sample) at baseline, and will recruit another 240 at endline.

Participant recruitment

For the purposes of this evaluation, a household is defined as a group of people who regularly cook together. Inclusion criteria for the household survey are:

For peer review only - http://bmjopen.bmj.com/site/about/guidelines.xhtml

- Household with someone who has delivered a baby within the past 12 months, irrespective of maternal or infant vital status
- Participant must be age 15 or older. If age 15-17, a legal guardian must be available for consent.
- Proxy participant (if woman deceased) must be over the age of 18
- Resident of the village identified for sampling (≥10 km from the facility)

To select a sample representative of women living at least 10km from our health facility, we employ multi-stage random sampling procedures (Figure 3). We begin the first stage of sampling by visiting every village within the catchment area of each study site, informing the local village leader of the purpose of the study and taking the GPS coordinates from the approximate geographical "center" of the village. We input these GPS coordinates into ArcGIS® Online (Esri, Redlands, CA) and use the line creation tool to draw the most direct route along the roads and paths visible on the World Imagery basemap between each village center and their associated health facility. We then use this network of roads to calculate the distance of each village to the health facility and develop a sampling frame of all villages within each catchment area located more than 10km from the health facility (rounding up from 9.5km). We then randomly select a sample of 10 villages from each catchment area with probability proportional to population size. We list every eligible village within a catchment area in Microsoft Excel® along with the total population of the village. We assign a series of numbers to each village, corresponding to the population size (i.e. if village 1 had 30 people, 1-30; village 2 had 20 inhabitants, 31-50), and use the random number generator function to select the villages in each catchment area.

Second, we work with community volunteers and village leaders to list all households within the selected villages that have a woman who had a delivery in the last year. We randomly order them by rolling a dice twice, first for a random start and then for a random skip until all households are ordered. We visit each household in that order and confirm their eligibility for study participation. We continue

For peer review only

down the list until six eligible household in each village are identified. We select additional villages and additional households if necessary to reach our sample of 2,400 households per round. This process assumes that the health facility staff are able to accurately and completely identify all villages within their catchment area.

INSERT FIGURE 3

The study team and community volunteers introduce the study to potential participants and request permission from the household head or most senior woman in the household to screen for eligibility. If household eligibility is confirmed, the study team proceeds with the informed voluntary consent process with the household head or senior woman. Once informed consent is obtained and documented from the household head or senior woman, the enumerator records the geo-location of the household and commences the interview or schedules a later appointment. The household head or senior woman responds to the first part of the survey for approximately 15 minutes, enumerating all of the people in the household in a table that captures demographics as well as recent deliveries and delivery outcomes.

Upon completion of the household demographics and enumeration, an eligible woman was selected to respond to the remainder of the survey. If more than one women in the household had delivered a baby in the past 12 months, the electronic data capture system is programmed to randomly selecte one eligible woman to respond to the remainder of the survey. The selected woman then consents separately, enrolls in the study, and completes the HHS in a private space where she feels comfortable. Completion of the HHS takes approximately 45 minutes.

Of the woman participants, 10% are randomly selected to participate in a 30-minute IDI immediately following the survey. IDI participants can take a short break after ther HHS, or reschedule if more convenient. The household-level sampling procedures described here have been conducted at

For peer review only

baseline (2016) and will be conducted at endline (2018) with a new cross-sectional sample of households and women within the households. Therefore, the same households are not followed over time.

Patient and public involvement

The development of the research question and outcome measures were informed by key stakeholders and patients' experience and preferences derived from free list responses, key informant interviews, and focus group discussions conducted during the formative evaluation [24–26]. Input from key stakeholders and community members helped to ensure that the design of the intervention would be responsive to community standards of acceptability of factors such as safety and comfort, and a feasible option to increase facility deliveries. Patients were not involved in studydesign, recruitment, and or conduct of the trial. Given the nature of the intervention, there was limited potential burden on patients and therefore, the burden of the randomized controlled trial was not assessed by the patients.

The primary audience for this evaluation is the Government of Zambia, particularly the Ministry of Health, Ministry of Community Development, and the Ministry of Chiefs and Traditional Affairs, which will use the results to inform the development of maternal and child health strategies and policies in Zambia. We have disseminated the baseline findings to key stakeholders internal to Zambia and will disseminate the full study findings after endline. Many of the findings will likely be of broader interest throughout the region and globally where maternal mortality is high, resources are low, and access to facility-based delivery remains an issue. As such, results of this evaluation will be disseminated as widely as possible through open-access journals, websites, and international conferences.

Procedures

Data collection

For peer review only

At baseline and endline, a local team of enumerators literate in the appropriate local language(s) and in English are trained in qualitative and quantitative research methods and human subjects' protection. Surveys are designed in SurveyCTO Collect software (Version 2.212; Dobility, Inc.) and are captured electronically using encrypted tablets. The IDIs are digitally captured on audio recorders. Enumerators explain the tablet system to all participants and explain the digital audio recorders to those selected for IDIs.

Several checks assure the quality of collected survey data. First, enumerators participate in an extensive 5-day training. Second, the enumerators are overseen by data collection team leads with greater experience in data collection fieldwork. Team leads are overseen by a field supervisor. Team leads and the field supervisors review surveys for accuracy and completeness nightly. Third, field supervisors randomly select a 5% subsample of households to be audited; the auditor revisits these households and repeats a subset of survey questions that are checked for reliability. Fourth, the field supervisors conduct a short nightly debrief with the data leads who each oversee three other data collectors and are responsible for conducting the IDIs. Debriefs cover the following topics: field challenges, sampling, total surveys conducted, and IDIs. Lastly, quantitative data are encrypted, uploaded and transferred nightly to the data analysis team where progress is reviewed in real time. On a nightly basis, qualitative data are removed from the recorders, saved on a password-protected computer, and tracked nightly.

Data management

Survey data are captured on tablets and saved to the internal memory. During each round of data collection, each evening, a data team supervisor reviews the survey and encrypts it so survey data are no longer accessible on the tablet. The supervisor uploads encrypted data nightly during the collection period to a secure server administered by SurveyCTO (Version 2.212; Dobility, Inc.). The

For peer review only

evaluation team downloads the encrypted data using the SurveyCTO Client software (Version 2.212; Dobility, Inc.), and decrypts the data using a decryption key generated by the research team.

The evaluation team oversees data entry, management, and storage for qualitative data. All IDIs are translated into English and transcribed verbatim. Digital recorders and paper copies of written notes are kept in a locked cabinet until transcriptions are checked for accuracy and completeness, at which point audio files are deleted and notes are shredded. The electronic transcriptions do not contain identifying information, only a study ID number linked to the quantitative survey. A separate linking file for the quantitative and qualitative data is password protected and only accessible to the study team.

Data analysis

The primary independent variable of interest is assignment to the intervention. For the analysis, we will compare baseline characteristics between the intervention and control groups to assess balance. We collect data on potential confounders to increase precision, analyze heterogeneity, and, if necessary, control for any potential imbalance between the groups.

The primary dependent variable is the probability of facility delivery for most recent birth, based on self-report by mothers. Secondary outcomes include:

- Use of MWHs for antenatal care, delivery or postnatal services
- Delivery by cesaerean section
- Maternal death
- Neonatal death

We initially considered other secondary morbidity outcomes, but because the data were self-reported and asked about experience up to 12 months before, there were limitations to what can reasonably be asked without introducing major recall bias. The survey captures additional indicators of

For peer review only - http://bmjopen.bmj.com/site/about/guidelines.xhtml

complications including IV antibiotics, blood transfusions, and referral to CEmONC, but we have limited secondary outcomes to those most likely to be clearly remembered.

All quantitative analyses will be conducted in SAS v9.4 (SAS, Cary, NC). Our quantitative analytic plan is threefold, yielding descriptive, bivariate and multivariate statistics. First, we will describe the study sample, stratifying by intervention and control group and testing for differences between the groups. Second, we will estimate differences between the groups for primary and secondary outcomes, controlling for a set of baseline demographics. . Categorical variables will be compared between the groups using a chi-squared test when cell sizes are sufficient or Fisher's exact test when the cell sizes are small; continuous variables will be compared using t-tests if normally distributed or non-parametric Wilcoxon rank sum tests if the distribution is non-normal. Third, we will fit several regression models to estimate the impact of the intervention on the primary and secondary outcomes, adjusting for baseline values, assignment matching variables, and any imbalanced covariates. To control for the phased timing of implementation, with MWHs opening at by including a variable in our main models that captures the month the home opened.

All qualitative data will be analyzed in Nvivo 10 © software (QSR International Pty Ltd.). We will conduct a content analysis of the IDI transcripts. Coding themes have been identified *a priori*. Additional themes will be included as they emerge. We will triangulate findings with the quantitative data to identify consistencies, inconsistencies or additional themes to be explored. We will use the themes developed during the baseline analysis to analyze the endline data and identify any new themes as they emerge.

To systematically assess confounders and the risk of bias at the pre-intervention phase, intervention phase, and post-intervention phase, we will use the ROBINS-I tool.[36] Guided by this tool, we will transparently report threats to validity of this quasi-experimental study during analysis,

For peer review only

interpretation and dissemination. Results for the primary and each secondary evaluation question will be presented.

ETHICS

Ethics approval and consent to participate

Ethical review boards

Prior to participant enrollment, ethical approvals were obtained from the Boston University Institutional Review Board (IRB), University of Michigan IRB (for a de-identified dataset only), and the ERES Converge Research IRB, a private local ethics board in Zambia. We also obtained official approval to proceed with the study from the Zambia National Health Research Authority, which is responsible for oversight of all research conducted in the country. Adverse events, unanticipated problems and any protocol changes will be reported to the IRBs and the Zambia National Health Research Authority per their guidelines, and all investigators will be informed.

Potential risks and protections

This study poses minimal risk to study participants and several steps were taken to minimize risk and burden. To reduce the risk of disclosure of personal or sensitive information enumerators are trained to stop participants from disclosing information that is too sensitive. Participation may cause some discomfort from answering certain questions, particularly if the maternal or neonatal health outcomes were adverse. Enumerators are trained to minimize any potential discomfort or harm to all participants during all study activities to the greatest extent possible. We minimize any waiting by participants by scheduling meetings during times convenient to participants and interviews are kept to as short of time as possible taking breaks if necessary.

For peer review only - http://bmjopen.bmj.com/site/about/guidelines.xhtml

Potential benefits

There are no direct individual benefits to participating in the study. The evaluation results will generate evidence on the impact of MWHs on facility delivery for those who live farthest away. Findings will provide insight for policy makers into how, if found to be effective, MWHs can be part of a broader strategy to improve maternal and neonatal health outcomes.

Participant confidentiality

Throughout the study, we take care to ensure the confidentiality of data obtained from study participants. The HHSs and IDIs are carried out in participants' private homes or somewhere the participant feels comfortable. We do not proceed with data collection until we can confirm that the location is acceptable and participants agree that they feel comfortable discussing study topics.

The linking file with identifiable data and basic demographics is stored in a separate file within the tablet system. Upon completion of data collection, all files are stored on a secure server during data analysis and report writing. Only BU/RTC investigators have access to identifiable data. All analyses by study partners are conducted on de-identified datasets per IRB approvals. Analyses are presented in aggregate format in technical reports to stakeholders and in manuscripts submitted for publication in scientific journals. Under no circumstances do organizations or individuals have access to the participant's individual demographic information and potential identifying information (job title, age range, sex, and village). As explained above, the qualitative data are de-identified, with basic demographics only.

Informed consent

Prior to any data collection, we discuss the purpose of the study with local leaders so that the study activities are clearly understood. If a household is eligible, the study team proceeds with the informed voluntary consent process from the household head or the most senior woman in the

For peer review only - http://bmjopen.bmj.com/site/about/guidelines.xhtml

household, introducing themselves, the purpose of the study, and explaining what we are asking of them in terms of participation, the risks and benefits, the right to withdraw without penalty at any time, that their information will be kept in a safe location, and that their answers will not be linked to their names. Participants are informed that the alternative is to not participate in the study. The study team slowly and clearly asks for consent to participate. If a selected household participant declines participation, the next household on the randomly ordered list of eligible households is contacted. If a household head or senior woman consents to participating, the study team docuements written informed consent and proceeds with the interview. In addition to the household head or a senior woman, using the same process we also consent the woman selected from within the household to respond to the survey; in some cases, this may be the same person. A maximum of two individuals are consented per household.

We anticipate about 15% of the sample in each round to be between 15-17 years of age. In Zambia, 'emancipated minors' can enroll if they provide assent and their guardian or husband also provides consent. If a woman's husband is 18 or older, then he can provide informed consent on behalf of his wife; however, if he is also under 18 years old, then her legal guardian must provide consent. If under 18, the research team will allow the woman to first determine if she wishes to join the study (assent is provided) and then obtain consent by the guardian or husband. Thus, the individual's wishes are protected and she can determine if she wishes to be part of the study.

All informed consent or assent/consent is documented with a signature; in the event a participant cannot write, a witness signs the informed consent. A participant retains a copy of the informed consent form. The informed consent and assent processes are always conducted in the language most preferred by the participant.

For peer review only

Costs and payments

For all activities, the participants volunteer only the time taken to complete this survey. There is no cash payment provided to participants for any portion of the study. Participants receive pieces of fabric as small tokens of appreciation in recognition of their time and opportunity costs, in line with local IRB procedures.

Limitations

This study has several limitations. First, half of study clusters could not be randomly assigned to either the intervention or control group due to political constraints and concern by the government about community fatigue. The selection bias resulting from the different assignment strategies is partially mitigated by ensuring comparison sites are matched on the same criteria as the other sites. Additionally, because one partner's comparison sites include existing MWHs as part of standard of care, and the other partner excluded sites with existing MWHs in , we will analyze the full sample as a quasi-experimental CBA study and we will estimate the impact in both the non-randomized and randomized subsamples to assess potential bias introduced by non-random assignment and the differences in comparison site selection. Second, we limited household eligibility to those living at least 10 km from the health facilities and will not be able to assess impact on women living nearer to facilities. However, remote women are the primary target of the MWH model and stand to benefit the most from the intervention. To manage this limitation, in separate process evaluation protocols, each partner is collecting facility-based data to understand any changes in demographics among those utilizing facilities for delivery using agreed upon indicators and data sources. Lastly, because there are two implementing partners, there is a risk that the MWH model will be implemented differently across the sites. To mitigate this risk, we have developed and agreed upon the precise elements of the MWH model based

For peer review only - http://bmjopen.bmj.com/site/about/guidelines.xhtml

on both partners' formative research [24–26] and will be assessing implementation fidelity using harmonized tools in the companion process evaluation protocols.

REFERENCES

1 The United Nations. Goal 3 .:. Sustainable Development Knowledge Platform. https://sustainabledevelopment.un.org/sdg3 (accessed 2 Feb 2018).

2 Central Statistical Office (CSO) Zambia, [Ministry of Health (MOH) [Zambia], ICF International. Zambia Demographic and Health Survey 2013-14. Rockville, Maryland, USA: 2014.

3 UN General Assembly. Resolution adopted by the General Assembly on 25 September 2015 70/1. Transforming our world: the 2030 Agenda for Sustainable Development. 2030. http://www.un.org/en/development/desa/population/migration/generalassembly/docs/globalcompact/A_RES_70_1_E.pdf (accessed 17 Jan 2018).

4 Republic of Zambia Ministry of Health. National Health Strategic Plan 2011 - 2015. 2011;:119.

5 Republic of Zambia Ministry of Health. Roadmap for Accelerating Reduction of Maternal , Newborn and Child Mortality , 2013-2016. 2016;:2013–6.

6 Gabrysch S, Cousens S, Cox J, *et al.* The influence of distance and level of care on delivery place in rural Zambia: a study of linked national data in a geographic information system. *PLoS Med* 2011;**8**. doi:10.1371/journal.pmed.1000394

7 Gabrysch S, Campbell OM, Lawn J, *et al.* Still too far to walk: Literature review of the determinants of delivery service use. *BMC Pregnancy Childbirth* 2009;**9**:34. doi:10.1186/1471-2393-9-34

8 Lohela TJ, Campbell OMR, Gabrysch S, *et al.* Distance to Care, Facility Delivery and Early Neonatal Mortality in Malawi and Zambia. *PLoS One* 2012;**7**:e52110. doi:10.1371/journal.pone.0052110

9 Sialubanje C, Massar K, Hamer DH, *et al.* Understanding the psychosocial and environmental factors and barriers affecting utilization of maternal healthcare services in Kalomo, Zambia: A qualitative study. *Health Educ Res* 2014;**29**:521–32. doi:10.1093/her/cyu011

10 Stekelenburg J, Van Lonkhuijzen L, Spaans W, *et al.* Maternity waiting homes in rural districts in Africa: A cornerstone of safe motherhood? *Curr Womens Health Rev* 2006;**2**:235–8. doi:10.2174/157340406778699914

11 Lori JR, Wadsworth AC, Munro ML, *et al.* Promoting access: The use of maternity waiting homes to achieve safe motherhood. *Midwifery* 2013;**29**:1095–102. doi:10.1016/j.midw.2013.07.020

12 Kelly J, Kohls E, Poovan P, *et al.* The role of a maternity waiting area (MWA) in reducing maternal mortality and stillbirths in high-risk women in rural Ethiopia. *BJOG* 2010;**117**:1377–83. doi:10.1111/j.1471-0528.2010.02669.x

13 Henry EG, Semrau K, Hamer DH, *et al.* The influence of quality maternity waiting homes on utilization of facilities for delivery in rural Zambia. *Reprod Health* 2017;**14**:68.

For peer review only - http://bmjopen.bmj.com/site/about/guidelines.xhtml

doi:10.1186/s12978-017-0328-z

14 Fogliati P, Straneo M, Mangi S, *et al.* A new use for an old tool: maternity waiting homes to improve equity in rural childbirth care. Results from a cross-sectional hospital and community survey in Tanzania. *Health Policy Plan* 2017;**32**:1354–60. doi:10.1093/heapol/czx100

15 García Prado A, Cortez R. Maternity waiting homes and institutional birth in Nicaragua: policy options and strategic implications. *Int J Health Plann Manage* 2012;**27**:150–66. doi:10.1002/hpm.1107

16 Chandramohan D, Cutts F, Millard P. The effect of stay in a maternity waiting home on perinatal mortality in rural Zimbabwe. *J Trop Med Hyg* 1995;**98**:261–7.http://www.ncbi.nlm.nih.gov/pubmed/7636923 (accessed 12 Dec 2016).

17 Chandramohan D, Cutts F, Chandra R. Effects of a maternity waiting home on adverse maternal outcomes and the validity of antenatal risk screening. *Int J Gynecol Obstet* 1994;**46**:279–84. doi:10.1016/0020-7292(94)90406-5

18 Gaym A, Pearson L, Soe KWW. Maternity waiting homes in Ethiopia--three decades experience. *Ethiop Med J* 2012;**50**:209–19.

19 Van Lonkhuijzen L, Stegeman M, Nyirongo R, *et al.* Use of Maternity Waiting Home in Rural Zambia. *Afr J Reprod Health* 2003;**7**:32–6. doi:10.1108/10650750910961929

20 Andemichael G, Haile B, Kosia A, *et al.* Maternity waiting homes: A panacea for maternal/neonatal conundrums in Eritrea. *J Eritrean Med Assoc* 2009;**4**:18–21. doi:10.4314/jema.v4i1.52112

21 van Lonkhuijzen L, Stekelenburg J, van Roosmalen J. Maternity waiting facilities for improving maternal and neonatal outcome in low-resource countries. *Cochrane database Syst Rev* 2012;**10**:CD006759. doi:10.1002/14651858.CD006759.pub3

22 Wild K, Barclay L, Kelly P, *et al.* The tyranny of distance: maternity waiting homes and access to birthing facilities in rural Timor-Leste. *Bull World Health Organ* 2012;**90**:97–103. doi:10.2471/BLT.11.088955

23 Ministry of Health (MOH) [Zambia]. Zambia National Health Strategic Plan 2017-2021. 2017.

24 Chibuye PS, Bazant ES, Wallon M, *et al.* Experiences with and expectations of maternity waiting homes in Luapula Province, Zambia: a mixed–methods, cross-sectional study with women, community groups and stakeholders. *BMC Pregnancy Childbirth* 2018;**18**:42. doi:10.1186/s12884-017-1649-1

25 Lori JR, Munro-Kramer ML, Mdluli EA, *et al.* Developing a community driven sustainable model of maternity waiting homes for rural Zambia. *Midwifery* 2016;**41**:89–95. doi:10.1016/j.midw.2016.08.005

26 Scott NA, Vian T, Kaiser JL, *et al.* Listening to the community: Using formative research to strengthen maternity waiting homes in Zambia. *PLoS One* 2018;**13**. doi:https://doi.org/10.1371/journal.pone.0194535

27 Central Statistical Office (CSO) Zambia. 2010 Census of Population and Housing - Southern Province Analytical Report. Lusaka: 2014. http://web0.psa.gov.ph/statistics/census/population-and-housing

28 Central Statistical Office (CSO) Zambia. 2010 Census of Population and Housing - Eastern Province Analytical Report. Lusaka: 2014.

For peer review only - http://bmjopen.bmj.com/site/about/guidelines.xhtml

https://www.zamstats.gov.zm/phocadownload/2010_Census/2010_Census_Analytical_Reports/Eastern Province Analytical Report - 2010 Census.pdf (accessed 19 Jan 2018).

29 Central Statistical Office (CSO) Zambia. Zambia 2010 Census of Population and Housing Population Summary Report. Lusaka: 2012. http://www.mcaz.gov.zm/wp-content/uploads/2014/10/2010-Census-of-Population-Summary-Report.pdf (accessed 19 Jan 2018).

30 Zambian Ministry of Health. The 2012 List of Health Facilities in Zambia: Preliminary Report, v15. 2013;:252.

31 SMGL. Saving Mothers Giving Life - Home. 2016.https://www.savingmothersgivinglife.org/ (accessed 16 Nov 2016).

32 Bernard HR. *Social Research Methods Qualitative and Quantitative Approaches*. 2nd ed. USA: : SAGE Publications, Inc 2013.

33 Hamer DH, Herlihy JM, Musokotwane K, *et al.* Engagement of the Community, Traditional Leaders, and Public Health System in the Design and Implementation of a Large Community-Based, Cluster-Randomized Trial of Umbilical Cord Care in Zambia. *Am J Trop Med Hyg* 2015;**92**:666–72. doi:10.4269/ajtmh.14-0218

34 Semrau KEA, Herlihy J, Grogan C, *et al.* Effectiveness of 4% chlorhexidine umbilical cord care on neonatal mortality in Southern Province, Zambia (ZamCAT): a cluster-randomised controlled trial. *Lancet Glob Heal* 2016;**4**:e827–36. doi:10.1016/S2214-109X(16)30215-7

35 Pagel C, Prost A, Lewycka S, *et al.* Intracluster correlation coefficients and coefficients of variation for perinatal outcomes from five cluster-randomised controlled trials in low and middle-income countries: results and methodological implications. *Trials* 2011;**12**:151. doi:10.1186/1745-6215-12-151

36 Sterne JA, Hernán MA, Reeves BC, *et al.* ROBINS-I: a tool for assessing risk of bias in non-randomised studies of interventions. *BMJ* 2016;**355**:i4919. doi:10.1136/BMJ.I4919

AUTHORS STATEMENT

NS led the scientific design, implementation, and was primarily responsible for drafting this manuscript. JK contributed to the development of the protocol, led the development of the study sample, coordinated data collection, and contributed to revisions of the manuscript. TV and RB contributed to the revisions and science of the protocol and data collection instruments. RF contributed to the development of the protocol, implemented the electronic data capture system, and contributed to the revisions of this manuscript. TN, GB, CB, and JL provided feedback on the protocol and reviewed and edited the final manuscript. DH provided scientific support, technical input into the survey design and critically reviewed and edited the final manuscript. PR helped conceptualize the scientific design of the

For peer review only - http://bmjopen.bmj.com/site/about/guidelines.xhtml

study, provided technical input into the survey design, sampling approach, and critically reviewed and edited the final manuscript. All authors read and approved the final version of the manuscript.

FUNDING

This program was developed and is being implemented in collaboration with *MSD for Mothers*, MSD's 10-year, $500 million initiative to help create a world where no woman dies giving life. *MSD for Mothers* is an initiative of Merck & Co., Inc., Kenilworth, N.J., U.S.A. (MRK 1846-06500.COL). The development of this article was additionally supported in part by the Bill & Melinda Gates Foundation (OPP1130329) https://www.gatesfoundation.org/How-We-Work/Quick-Links/Grants-Database/Grants/2015/07/OPP1130329 and The ELMA Foundation (ELMA-15-F0017) http://www.elmaphilanthropies.org/the-elma-foundation/. The funders had no role in study design, data collection and analysis, decision to publish, or preparation of the manuscript. The content is solely the responsibility of the authors and does not reflect positions or policies of MSD, the Bill & Melinda Gates Foundation or The ELMA Foundation.

CONFLICTS OF INTEREST STATEMENT

The authors declare that they have no competing interests.

FIGURE LEGEND

Figure 1. Minimum Maternity Waiting Home Model in the Maternity Home Alliance for Intervention Sites (n=20).

Figure 2. Map of the Maternity Home Alliance Intervention and Control Study Sites by Partner

Figure 3. Multi-stage Random Sampling Strategy for Baseline and Endline

For peer review only - http://bmjopen.bmj.com/site/about/guidelines.xhtml

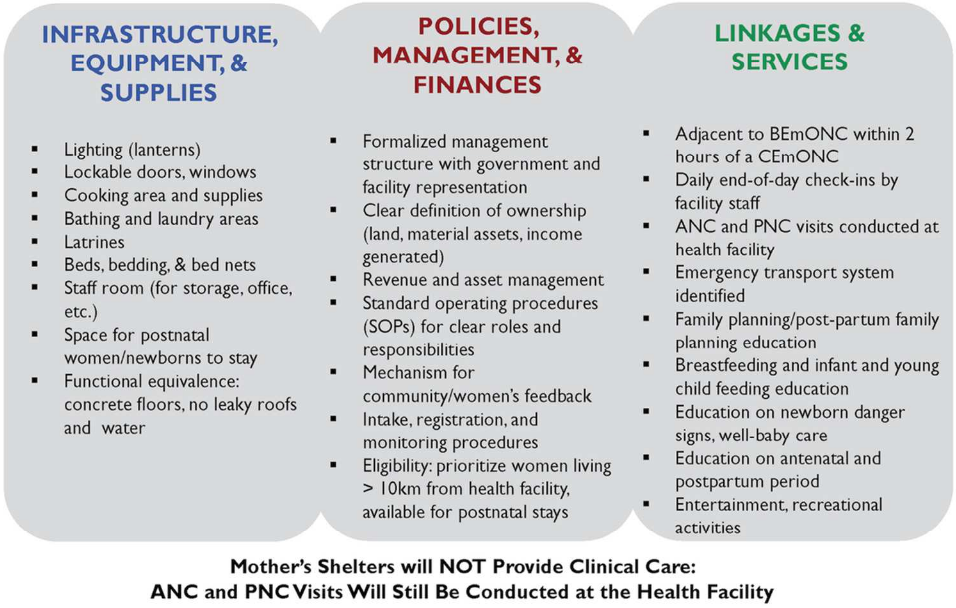

Figure 1. Minimum Maternity Waiting Home Model in the Maternity Home Alliance for Intervention Sites (n=20).

101x66mm (300 x 300 DPI)

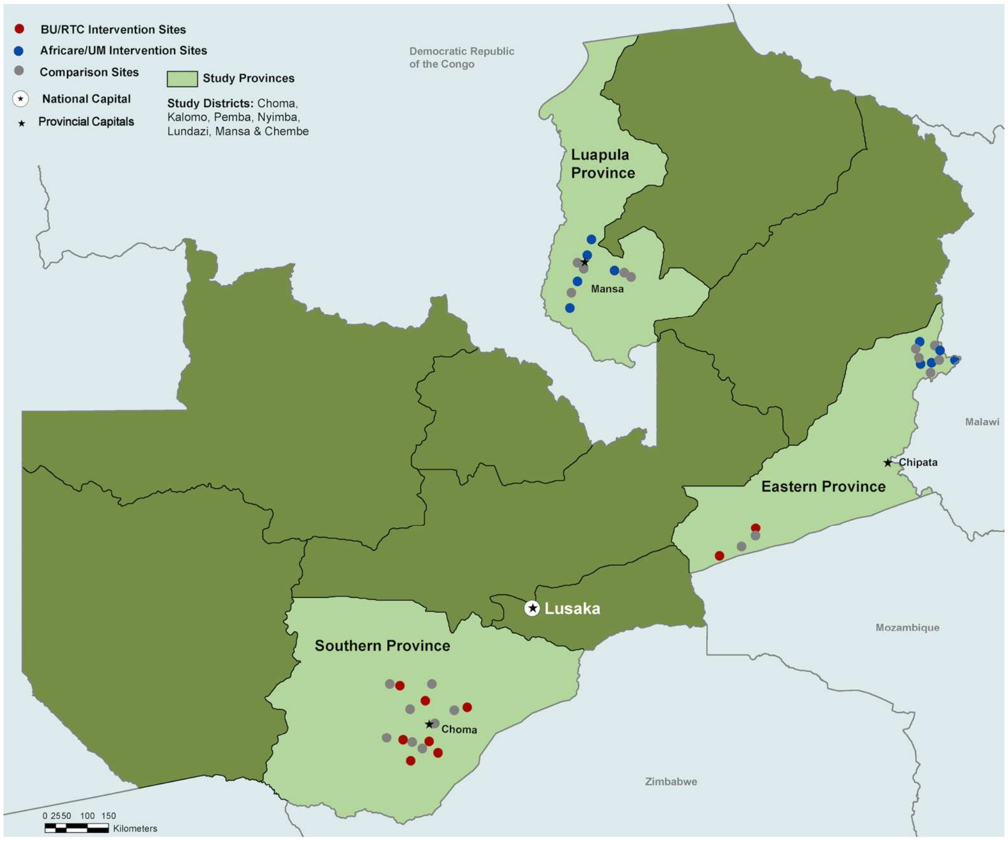

Figure 2. Map of the Maternity Home Alliance Intervention and Control Study Sites by Partner

173x144mm (300 x 300 DPI)

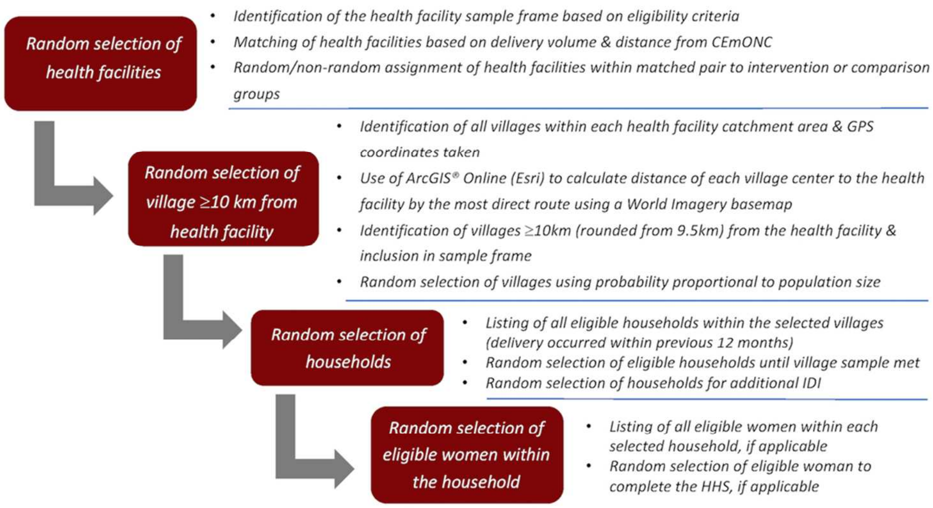

Figure 3. Multi-stage Random Sampling Strategy for Baseline and Endline

97x54mm (300 x 300 DPI)

For peer review only

STANDARD PROTOCOL ITEMS: RECOMMENDATIONS FOR INTERVENTIONAL TRIALS

SPIRIT 2013 Checklist: Recommended items to address in a clinical trial protocol and related documents*

Section/item	Item No	Description	Addressed on page number
**Administrative information**			
Title	1	Descriptive title identifying the study design, population, interventions, and, if applicable, trial acronym	_____1________
Trial registration	2a	Trial identifier and registry name. If not yet registered, name of intended registry	_3,clinicaltrials.gov
	2b	All items from the World Health Organization Trial Registration Data Set	1-23 and on clinicaltrials.gov
Protocol version	3	Date and version identifier	______na_____
Funding	4	Sources and types of financial, material, and other support	______23_____
Roles and responsibilities	5a	Names, affiliations, and roles of protocol contributors	______1_______
	5b	Name and contact information for the trial sponsor	______23______
	5c	Role of study sponsor and funders, if any, in study design; collection, management, analysis, and interpretation of data; writing of the report; and the decision to submit the report for publication, including whether they will have ultimate authority over any of these activities	______23_______

For peer review only - http://bmjopen.bmj.com/site/about/guidelines.xhtml

	5d	Composition, roles, and responsibilities of the coordinating centre, steering committee, endpoint adjudication committee, data management team, and other individuals or groups overseeing the trial, if applicable (see Item 21a for data monitoring committee)	____na_________
**Introduction**			
Background and rationale	6a	Description of research question and justification for undertaking the trial, including summary of relevant studies (published and unpublished) examining benefits and harms for each intervention	_____5,6______
	6b	Explanation for choice of comparators	______8_______
Objectives	7	Specific objectives or hypotheses	_____5,6_______
Trial design	8	Description of trial design including type of trial (eg, parallel group, crossover, factorial, single group), allocation ratio, and framework (eg, superiority, equivalence, noninferiority, exploratory)	_____8________
**Methods: Participants, interventions, and outcomes**			
Study setting	9	Description of study settings (eg, community clinic, academic hospital) and list of countries where data will be collected. Reference to where list of study sites can be obtained	_____7________
Eligibility criteria	10	Inclusion and exclusion criteria for participants. If applicable, eligibility criteria for study centres and individuals who will perform the interventions (eg, surgeons, psychotherapists)	_____8,9,12____
Interventions	11a	Interventions for each group with sufficient detail to allow replication, including how and when they will be administered	______6_______
	11b	Criteria for discontinuing or modifying allocated interventions for a given trial participant (eg, drug dose change in response to harms, participant request, or improving/worsening disease)	______na_______
	11c	Strategies to improve adherence to intervention protocols, and any procedures for monitoring adherence (eg, drug tablet return, laboratory tests)	_____na________
	11d	Relevant concomitant care and interventions that are permitted or prohibited during the trial	______na_______

For peer review only - http://bmjopen.bmj.com/site/about/guidelines.xhtml

Outcomes	12	Primary, secondary, and other outcomes, including the specific measurement variable (eg, systolic blood pressure), analysis metric (eg, change from baseline, final value, time to event), method of aggregation (eg, median, proportion), and time point for each outcome. Explanation of the clinical relevance of chosen efficacy and harm outcomes is strongly recommended	____6,7,16______
Participant timeline	13	Time schedule of enrolment, interventions (including any run-ins and washouts), assessments, and visits for participants. A schematic diagram is highly recommended (see Figure)	_____11_______
Sample size	14	Estimated number of participants needed to achieve study objectives and how it was determined, including clinical and statistical assumptions supporting any sample size calculations	_____11________
Recruitment	15	Strategies for achieving adequate participant enrolment to reach target sample size	_____13________

Methods: Assignment of interventions (for controlled trials)

Allocation:

Sequence generation	16a	Method of generating the allocation sequence (eg, computer-generated random numbers), and list of any factors for stratification. To reduce predictability of a random sequence, details of any planned restriction (eg, blocking) should be provided in a separate document that is unavailable to those who enrol participants or assign interventions	____9, 12, 13, __
Allocation concealment mechanism	16b	Mechanism of implementing the allocation sequence (eg, central telephone; sequentially numbered, opaque, sealed envelopes), describing any steps to conceal the sequence until interventions are assigned	______na_______
Implementation	16c	Who will generate the allocation sequence, who will enrol participants, and who will assign participants to interventions	_____na________
Blinding (masking)	17a	Who will be blinded after assignment to interventions (eg, trial participants, care providers, outcome assessors, data analysts), and how	______na_______
	17b	If blinded, circumstances under which unblinding is permissible, and procedure for revealing a participant's allocated intervention during the trial	______na_______

Methods: Data collection, management, and analysis

Data collection methods	18a	Plans for assessment and collection of outcome, baseline, and other trial data, including any related processes to promote data quality (eg, duplicate measurements, training of assessors) and a description of study instruments (eg, questionnaires, laboratory tests) along with their reliability and validity, if known. Reference to where data collection forms can be found, if not in the protocol	____6-14_______
	18b	Plans to promote participant retention and complete follow-up, including list of any outcome data to be collected for participants who discontinue or deviate from intervention protocols	_____na________
Data management	19	Plans for data entry, coding, security, and storage, including any related processes to promote data quality (eg, double data entry; range checks for data values). Reference to where details of data management procedures can be found, if not in the protocol	____14, 15______
Statistical methods	20a	Statistical methods for analysing primary and secondary outcomes. Reference to where other details of the statistical analysis plan can be found, if not in the protocol	_____15, 16_____
	20b	Methods for any additional analyses (eg, subgroup and adjusted analyses)	______16_______
	20c	Definition of analysis population relating to protocol non-adherence (eg, as randomised analysis), and any statistical methods to handle missing data (eg, multiple imputation)	______na_______

Methods: Monitoring

Data monitoring	21a	Composition of data monitoring committee (DMC); summary of its role and reporting structure; statement of whether it is independent from the sponsor and competing interests; and reference to where further details about its charter can be found, if not in the protocol. Alternatively, an explanation of why a DMC is not needed	_____na________
	21b	Description of any interim analyses and stopping guidelines, including who will have access to these interim results and make the final decision to terminate the trial	______na_______
Harms	22	Plans for collecting, assessing, reporting, and managing solicited and spontaneously reported adverse events and other unintended effects of trial interventions or trial conduct	______16, 17____
Auditing	23	Frequency and procedures for auditing trial conduct, if any, and whether the process will be independent from investigators and the sponsor	______na_______

Ethics and dissemination

For peer review only - http://bmjopen.bmj.com/site/about/guidelines.xhtml

Research ethics approval	24	Plans for seeking research ethics committee/institutional review board (REC/IRB) approval	______16_______
Protocol amendments	25	Plans for communicating important protocol modifications (eg, changes to eligibility criteria, outcomes, analyses) to relevant parties (eg, investigators, REC/IRBs, trial participants, trial registries, journals, regulators)	______16, 17____
Consent or assent	26a	Who will obtain informed consent or assent from potential trial participants or authorised surrogates, and how (see Item 32)	____18, 19______
	26b	Additional consent provisions for collection and use of participant data and biological specimens in ancillary studies, if applicable	_____na________
Confidentiality	27	How personal information about potential and enrolled participants will be collected, shared, and maintained in order to protect confidentiality before, during, and after the trial	____17, 18______
Declaration of interests	28	Financial and other competing interests for principal investigators for the overall trial and each study site	________23______
Access to data	29	Statement of who will have access to the final trial dataset, and disclosure of contractual agreements that limit such access for investigators	_____18________
Ancillary and post-trial care	30	Provisions, if any, for ancillary and post-trial care, and for compensation to those who suffer harm from trial participation	_____na________
Dissemination policy	31a	Plans for investigators and sponsor to communicate trial results to participants, healthcare professionals, the public, and other relevant groups (eg, via publication, reporting in results databases, or other data sharing arrangements), including any publication restrictions	______19_______
	31b	Authorship eligibility guidelines and any intended use of professional writers	_____22, 23_____
	31c	Plans, if any, for granting public access to the full protocol, participant-level dataset, and statistical code	______na_______
**Appendices**			
Informed consent materials	32	Model consent form and other related documentation given to participants and authorised surrogates	_Can be provided upon request__

For peer review only - http://bmjopen.bmj.com/site/about/guidelines.xhtml

Biological specimens	33	Plans for collection, laboratory evaluation, and storage of biological specimens for genetic or molecular analysis in the current trial and for future use in ancillary studies, if applicable	_____na________

*It is strongly recommended that this checklist be read in conjunction with the SPIRIT 2013 Explanation & Elaboration for important clarification on the items. Amendments to the protocol should be tracked and dated. The SPIRIT checklist is copyrighted by the SPIRIT Group under the Creative Commons "Attribution-NonCommercial-NoDerivs 3.0 Unported" license.

For peer review only

For peer review only - http://bmjopen.bmj.com/site/about/guidelines.xhtml

BMJ Open

Impact of maternity waiting homes on facility delivery among remote households in Zambia: protocol for a quasi-experimental, mixed-methods study

Journal:	*BMJ Open*
Manuscript ID	bmjopen-2018-022224.R2
Article Type:	Protocol
Date Submitted by the Author:	12-Jul-2018

Complete List of Authors:	Scott, Nancy; Boston University School of Public Health, Global Health<br>Kaiser, Jeanette; Boston University School of Public Health, Global Health<br>Vian, Taryn; Boston University School of Public Health, Global Health<br>Bonawitz, Rachael; Boston University School of Public Health, Global Health; Boston University School of Medicine, Pediatrics<br>Fong, Rachel; Boston University School of Public Health, Global Health<br>Ngoma, Thandiwe; Right to Care Zambia<br>Biemba, Godfrey; National Health Research Authority, Pediatric Centre of Excellence<br>Boyd, Carol; University of Michigan School of Nursing, Health Behavior & Biological Sciences<br>Lori, Jody; University of Michigan School of Nursing, Center for Global Affairs & PAHO/WHO Collaborating Center<br>Hamer, D.; Boston University,<br>Rockers, Peter; Boston University, Department of Global Health

**Primary Subject Heading**:	Obstetrics and gynaecology
Secondary Subject Heading:	Research methods, Global health, Qualitative research
Keywords:	maternity waiting home, maternal health, skilled birth attendance, mixed methods, impact evaluation, Zambia

SCHOLARONE™ Manuscripts

Title: *Impact of maternity waiting homes on facility delivery among remote households in Zambia: protocol for a quasi-experimental, mixed-methods study*

Authors:

Name	Institutional Address	Email Address
Nancy Scott	Department of Global Health, Boston University School of Public Health, Boston, USA<br>801 Massachusetts Ave, Boston, MA 02118	nscott@bu.edu (corresponding)<br>Tel: +1-617-414-1278
Jeanette Kaiser	Department of Global Health, Boston University School of Public Health<br>Boston, MA USA	jlkaiser@bu.edu
Taryn Vian	Department of Global Health, Boston University School of Public Health<br>Boston, MA USA	tvian@bu.edu
Rachael Bonawitz	Department of Global Health, Boston University School of Public Health<br>Boston, MA USA<br>Department of Pediatrics, Boston University School of Medicine<br>Boston, MA USA	rebonawi@bu.edu
Rachel Fong	Department of Global Health, Boston University School of Public Health<br>Boston, MA USA	rmfong@bu.edu
Thandiwe Ngoma	Right to Care-Zambia<br>Lusaka, Zambia	ngoma.thandiwe@gmail.com
Godfrey Biemba	National Health Research Authority, Pediatric Centre of Excellence<br>Lusaka, Zambia	gbiemba@gmail.com
Carol J. Boyd	Department of Health Behavior & Biological Sciences<br>School of Nursing, University of Michigan<br>Ann Arbor, MI USA	caroboyd@med.umich.edu
Jody R. Lori	Center for Global Affairs &<br>PAHO/WHO Collaborating Center<br>School of Nursing, University of Michigan<br>Ann Arbor, MI USA	jrlori@umich.edu
Davidson H. Hamer	Department of Global Health, Boston University School of Public Health<br>Boston, MA USA<br><br>Section of Infectious Diseases, Department of Medicine, Boston Medical Center<br>Boston, MA USA	dhamer@bu.edu
Peter C. Rockers	Department of Global Health, Boston University School of Public Health<br>Boston, MA USA	prockers@bu.edu

Word count: 4,200 (excluding title page, abstract, references, figures and tables)

For peer review only - http://bmjopen.bmj.com/site/about/guidelines.xhtml

List of Acronyms

Africare/UM	Africare/University of Michigan
ANC	Antenatal care
BEmONC	Basic emergency obstetric and newborn care
BU/RTC	Boston University/Right to Care Zambia
CBA	Controlled before-and-after
CEmONC	Comprehensive emergency obstetric and newborn care
GRZ	Government of the Republic of Zambia
HHS	Household survey
IDI	In-depth interview
IRB	Institutional review board
MHA	Maternity Homes Alliance
MMR	Maternal mortality ratio
MSD	Merck Sharp & Dohme
MWH	Maternity Waiting Home
PNC	Postnatal care
SDG	Sustainable Development Goals

For peer review only

ABSTRACT

Introduction: Maternity waiting homes (MWHs) aim to improve access to facility delivery in rural areas. However, there is limited rigorous evidence of their effectiveness. Using formative research, we developed a MWH intervention model with three components: infrastructure, management, and linkage to services. This protocol describes a study to measure the impact of the MWH model on facility delivery among women living farthest (≥10km) from their designated health facility in rural Zambia. This study will generate key new evidence to inform decision making for MWH policy in Zambia and globally.

Methods and analysis: We are conducting a mixed-methods quasi-experimental impact evaluation of the MWH model using a controlled before-and-after design in 40 health facility clusters. Clusters were assigned to the intervention or control group using two methods: 20 clusters were randomly assigned using a matched-pair design; the other 20 were assigned without randomization due to local political constraints. Overall, 20 study clusters receive the MWH model intervention while 20 control clusters continue to implement the 'standard of care' for waiting mothers. We recruit a repeated cross-section of 2,400 randomly sampled recently-delivered women at baseline (2016) and endline (2018); all participants are administered a household survey and a 10% subsample also participates in an in-depth interview. We will calculate descriptive statistics and adjusted odds ratios; qualitative data will be analyzed using content analysis. The primary outcome is the probability of delivery at a health facility; secondary outcomes include utilization of MWHs and maternal and neonatal health outcomes.

Ethics and dissemination: Ethical approvals were obtained from the Boston University Institutional Review Board (IRB), University of Michigan IRB (de-identified data only), and the ERES Converge IRB in Zambia. Written informed consent is obtained prior to data collection. Results will be disseminated to key stakeholders in Zambia, then through open-access journals, websites, and international conferences.

Trial Registration: ClinicalTrials.gov Identifier: NCT02620436

Keywords: maternity waiting home, maternal health, facility delivery, mixed methods, impact evaluation, Zambia

For peer review only

ARTICLE SUMMARY

Strengths and limitations of this study

This study has several strengths and limitations, including:

- To the best of our knowledge, this is the first large-scale impact evaluation of MWHs, employing a rigorous controlled before-and-after, quasi-experimental design and using mixed-methods.
- For generalizability, a representative sample of recently-delivered women living most remotely is selected using a multi-stage, random sampling strategy for both the quantitative household surveys and the qualitative in-depth interviews.
- Half of study clusters could not be randomly assigned to either the intervention or control group due to political constraints, resulting in quasi-experimental study design.
- Because remote women stand to benefit the most from the MWH model, eligibility is limited to those living at least 10km from the health facilities; findings will therefore not be able to assess impact of the intervention on women living nearer to facilities.
- In companion protocols, implementation fidelity of the core elements of the MWH model is assessed by each partner using harmonized tools.

For peer review only

INTRODUCTION

The Sustainable Development Goals (SDGs) include a target of reducing the global maternal mortality ratio (MMR) to less than 70 deaths per 100,000 live births by 2030.[1] Zambia's MMR is currently 398 deaths per 100,000 live births, well above the SDG target.[2,3] Skilled care at every birth, one of the two SDG indicators for MMR, is recommended. What remains unanswered is how to best facilitate access to intrapartum and postpartum care, particularly in rural and remote areas where distance and poor transportation severely restrict access to care. The Government of the Republic of Zambia (GRZ) is committed to improving maternal health and encourages facility-based delivery for all women,[4,5] though accessing facilities for birth is challenging for women living in remote areas.[6–9]

Maternity waiting homes (MWHs) are lodgings located near health facilities where mothers who are close to term can await delivery. These homes are meant to provide pregnant women with the option of planning ahead and traveling to health facilities well before labor begins. MWHs may be a promising strategy to improve access to facilities for delivery, the evidence is mixed. While some evidence suggests they are associated with higher rates of facility delivery and improved maternal health outcomes,[10–20] a Cochrane review found that there are no randomized or quasi-randomized trials assessing the effectiveness of MWHs in low-resource settings.[21] Additionally, it is unclear if MWHs can increase access to facility delivery among women living most remotely.[19,22] Rigorous evidence on the impact of MWHs on facility deliveries is needed.

This protocol describes a study being conducted by the Maternity Homes Alliance (MHA), a partnership between the GRZ, Boston University and Right to Care Zambia, formerly the Zambian Center for Applied Health Research and Development (BU/RTC), Africare and the University of Michigan (Africare/UM), and funded by Merck Sharp and Dohme (MSD) for Mothers, the Bill & Melinda Gates Foundation, and The ELMA Foundation. The MHA hypothesizes that MWHs can remove the distance

For peer review only - http://bmjopen.bmj.com/site/about/guidelines.xhtml

barrier and increase access to facility-based delivery. In this study, we test the impact of MWHs on facility delivery among women living at least 10km from health facilities in rural Zambia.

MWHs have the potential to improve access to facility delivery, particularly for women in rural areas living far from health facilities. Despite their widespread use in developing countries, there is currently little evidence of MWH effectiveness. Using community input, we developed a MWH model and are evaluating it for impact. To the best of our knowledge, this is the first large-scale impact evaluation of MWHs. Findings will generate evidence surrounding the effectiveness of MWHs on improving facility deliveries for remote populations in Zambia and other countries with similar rural and highly dispersed populations.

Intervention

While the government of Zambia supports the use of MWHs as a strategic method to increase access to skilled birth attendance [5,23] and MWHs have existed in Zambia for decades, there is no specific policy or plan for the scale-up of MWHs and their general quality remains low.[13,24–26] MWHs have been largely constructed through community initiatives or international donors, with limited support for their long-term maintenance.[24–26] Formative evaluations conducted previously by members of the study team in the current study setting showed that MWHs could be an acceptable and feasible option to improve access to facilities for delivery.[24–26] Informed by these findings, the core MWH model was designed to be responsive to community expectations, community-defined standards of acceptability and their perceptions of quality including safety, comfort, management and services offered (Figure 1). In direct response to the formative data, the model includes the following:

- **Infrastructure, Supplies and Equipment:** The core MWH model has concrete walls and floors, roofs that do not leak, latrines, a private bathing space, water within a reasonable distance, a covered cooking space, and storage space. For safety, the core MWH model has lockable doors,

For peer review only

windows, cupboards, and lighting. Amenities include beds, mattresses, bedding, mosquito nets, and cooking utensils.

- **Policies, Management and Finances:** The core MWH model is community-owned and operated, as requested by the Ministry of Health. The policies, management, and financial structures are adaptable to site-specific needs and preferences, though all have a formalized governance and management structure with community, government and health facility representation. Each also has a management unit responsible for daily operations including registering and orienting women, record keeping and maintenance.
- **Linkages & Services:** Each core MWH model is situated close to the health facility to ensure timely access to clinical care when a woman's labor begins. A health facility staff provides daily check-ins with waiting women, though clinical care visits continue to be conducted at the health facility, not in the MWH. Women staying at the MWH have the opportunity to participate in maternal and child education courses offered by the health facility staff or community health workers.

INSERT FIGURE 1

The core MWH model is promoted in the community through several mechanisms. First, health facility staff promote the MWH at all ANC visits. Over 95% of women attend at least the first ANC visit , so most women are exposed at the health facility.[2] Second, Safe Motherhood Action Group members promote the use of MWHs during their routine outreach activities. Lastly, the traditional leadership (chiefs and headmen) actively promotes the use of MWHs at their community meetings. The MWH model targets all pregnant women within 1-2 weeks of their estimated delivery date resident within the

For peer review only - http://bmjopen.bmj.com/site/about/guidelines.xhtml

catchment area, prioritizing those women living farthest away (i.e. > 10 km from the health facility). The 20 MWHs opened in phases between mid-2016 and mid-2017.

METHODS

Evaluation questions

The primary research question is:

1. What is the impact of the MWH model on the probability of facility delivery among mothers living more than 10 km from the health facility?

Secondary questions include:

1. Do awareness and perceptions of health facility-associated safe delivery and health facility delivery intention among pregnant women living in communities located more than 10 km from the health facility change over time in MWH model sites?
2. How do awareness and perceptions of MWHs by communities located more than 10 km from the health facility change over the period of this study?
3. What financial impact does the use of the MWH model have on the families of women who utilize it?
4. How does the perception of quality and acceptability differ between MWH model sites and comparison sites?
5. What is the impact of the MWH model on maternal and neonatal health outcomes among those living more than 10 km from the facility?

Study setting

This study began in March 2016 and will be completed in December 2018. The intervention and comparison sites are located in the primarily rural Zambian districts of Choma, Kalomo and Pemba

For peer review only - http://bmjopen.bmj.com/site/about/guidelines.xhtml

Districts of Southern Province; Nyimba and Lundazi Districts of Eastern Province; and Mansa and Chembe Districts of Luapula Province (Figure 2).

INSERT FIGURE 2

Choma district has a population of 247,860 and a population density of 34/km^2, with 68.7% of its population being rural. Kalomo district has a population of 258,570 and a population density of 17.2/km^2, with 91.8 percent of its population being rural.[27] Nyimba district has a population of 85,025 and a population density of 8.1/km^2, with 91 percent of its population being rural. Lundazi district has a population of 323,870 and a population density of 23/km^2, with 95.1 percent of its population being rural.[28] Mansa district has a population of 228,392 and a population density of 23.1/km^2, with 61.9 percent of its population being rural.[29]

Study design

This study employs a quasi-experimental controlled before-and-after (CBA) design with a total of 40 study clusters, 20 intervention and 20 control clusters. Clusters consist of health facilities and their catchment households. Intervention clusters are receiving the MWH model, inclusive of newly constructed homes with the elements from the three domains: (1) infrastructure, equipment and supplies; 2) policies, management and finances; and 3) linkages and services detailed in the intervention section of the protocol. Control clusters are implementing the 'standard of care' for waiting mothers in Zambia. Because no national policy exists, the standard of care is facility-driven and varies widely. Some standard-of-care facilities have no designated space for a mother to wait; others have no MWH but provide a designated space for waiting mothers within the clinic; and a small number have an existing MWH-like structure but with highly variable quality.[13]

For peer review only - http://bmjopen.bmj.com/site/about/guidelines.xhtml

Eligibility criteria of study clusters

Because the intervention aims to generate demand for health facility delivery, it is critical that facilities are capable of managing basic emergency obstetric and neonatal complications (BEmONC). Because of inconsistencies in available secondary data sources across the different districts, we established supplemental criteria that could be drawn from the available sources.[30,31] Clusters were eligible for inclusion in the study if the health facility was located ≤2 hours driving time to a comprehensive emergency obstetric and neonatal care (CEmONC) capable referral facility, performed a minimum of 150 deliveries per year AND met at least one of two sets of conditions below:

Eligibility condition set 1:

i. Facility is able to provide at least 5 of 7 BEmONC signal functions based on 2015 data;

Eligibility condition set 2:

i. Facility has at least one skilled birth attendant on staff;
ii. Facility routinely provide active management of third stage of labor;
iii. Facility has had no stock outs of oxytocin in the last 12 months;
iv. Facility has had no stock outs of magnesium sulfate in the last 12 months;

Selection and assignment of study clusters to study arm

There is a total of 40 clusters (20 intervention, 20 comparison) in this study (Table 1). Each implementing partner used different methods to select and assign clusters to study arms. One partner had a total of 36 eligible health facilities that were located ≤2 hours driving time to a referral facility and performed a minimum of 150 deliveries. Of those, 22 (61%) met one of the two eligibility conditions. This partner selected the 20 farthest away from referral, created 10 pairs matched on annual delivery volume and distance, then randomized matched pairs to intervention or control, using the RAND

For peer review only - http://bmjopen.bmj.com/site/about/guidelines.xhtml

function in Microsoft Excel®. Sites in each arm were included, regardless if it had an existing infrastructure or space that functioned as a MWH, and considered standard of care. Those with existing infrastructure were not structurally sound.

Table 1: Quasi-experimental study design to evaluate the impact of MWHs

Randomized subsample (n=20 clusters)	Non-randomized subsample (n=20 clusters)	Non-randomized full sample (n=40 clusters)
R O1 X O2	NR O1 X O2	NR O1 X O2
R O1 _ O2	NR O1 _ O2	NR O1 _ O2

X = Minimum Core Maternity Home (see above)
O = Observations at baseline (O1, in 2016) and endline (O2, in 2018) at intervention (X) and comparison (_) sites.
R = cluster randomized; NR = not randomized

The other partner had a total of 29 eligible health facilities that were located ≤2 hours driving time to a referral facility and performed a minimum of 150 deliveries. Of those, 22 (76%) met one of the two sets of eligibility conditions. This partner was unable to randomly allocate sites to a study arm due to local political constraints, as the Ministry of Health feared community fatigue due to the large number of organizations implementing projects and conducting research. As such, the partner worked with the Ministry of Health to identify 10 intervention sites using the same eligibility critera. They then selected comparison sites, matched to intervention sites on annual delivery volume and distance to a referral hospital. Sites with an existing infrastructure that functioned as a MWH, were not considered as an option for comparison sites. After selecting sites, both partners then constructed the core MWH model at each of the 20 intervention sites.

Data sources

Population data are being collected from two main sources: household surveys (HHS) and in-depth interviews (IDIs). Baseline data collection occurred in early 2016 prior to the implementation of the MWH model in intervention clusters; endline data collection will occur in late 2018, after an 18-month intervention period. The HHS is administered to a sample of 2,400 recently delivered women

For peer review only

(eligibility criteria described below) residing in intervention and control clusters. In the case of maternal death, the household head or senior woman was interviewed as a proxy participant.

The HHS captures information on the domains and data fields seen in Table 2. The HHS was pretested among a sample of 50 participants representing all the major local languages. At baseline, only small adjustments were made in response to the pre-test, primarily changing formal translations into the vernacular.

Table 2. Summary table of data fields collected from the household survey

**Household Panel:**	• Geo-coordinates of household/distance from nearest health facility<br>• Age and sex of household members<br>• Education level of household members<br>• Recent pregnancy/delivery of household members
**Individual Demographics and Household Characteristics**	• Number of pregnancies<br>• Outcome of pregnancies<br>• Number of living/deceased children<br>• Characteristics of living quarters (e.g., roof type, floor type, cooking fuel type)<br>• Access to and quality of water<br>• Household wealth indicators and assets
**Last Pregnancy**	• Antenatal care services utilized<br>• HIV testing<br>○ Status<br>○ PMTCT*<br>• Perceived satisfaction with antenatal care
**Last Delivery**	• Location of last delivery<br>○ Decision-maker in location of delivery<br>○ Mode of transportation<br>• Referral and bypassing<br>• Receipt of CEmONC services (C-section, blood transfusion, IV antibiotics)<br>• Perceived quality/satisfaction with delivery services<br>• Maternal and neonatal vital status
**Use of MWH**	• Knowledge of MWH<br>• Source of knowledge of MWH<br>• Nearest MWH to home<br>• Use of MWH before/after last delivery<br>○ Cost of using mother's shelter<br>○ Perceived quality of mother's shelter (safety, comfort, management and services)<br>○ Satisfaction with mother's shelter

For peer review only - http://bmjopen.bmj.com/site/about/guidelines.xhtml

	• Prior use of MWH<br>• Intended future use of MWH
**Cost of Delivery and Delivery Planning**	• Planned or intended location for delivery<br>○ Adherence to planned or intended location for delivery<br>• Barriers to birth plan adherence<br>• Savings for last delivery<br>• Cost of last delivery (broken down by expense)
**Postnatal Care (PNC) and infant health**	• Time to first maternal and newborn post-natal visit after delivery<br>• Perceived quality of post-natal services received<br>• Breastfeeding practices<br>• Supplementary feeding practices<br>• Newborn vaccination status<br>• PMTCT/ART[++] for newborn<br>• Interactions between the parent and the child<br>• Maternal depression assessment<br>• Health seeking behavior for child's last illness
**Health Care Knowledge and Beliefs**	• Use of contraceptives for family planning<br>• Primary barriers to accessing health care<br>○ Primary barriers to accessing skilled delivery services

[*]PMTCT = Prevention of mother-to-child transmission of HIV; [++]ART = Antiretroviral therapy

In-depth interviews are conducted among a subsample of 240 HHS participants in order to gain a deeper understanding of community awareness, perceptions and experiences. Because the seven districts are spread out and culturally different, we wanted to ensure we reached saturation or predictability in each district to better explore context with the qualitative data.[32] Consequently, we planned to conduct a large number of in-depth interviews to make sure there was sufficient coverage of different populations to provide insight into the quantitative survey findings. IDI content builds upon themes captured in the HHS and includes perceptions of labor and delivery practices, barriers to accessing care, knowledge and awareness of MWHs, sources of knowledge of MWH, perceptions of the quality of maternity homes (safety, comfort, management and services), perceptions of MWH ownership, perceptions of health facility, and expenses incurred for last delivery.

The population-based approach captures the experiences of those who utilized the facility in their catchment, other facilities, and those who did not access a facility for delivery, allowing us to more

For peer review only

accurately estimate the impact of the MWH model intervention among women living farthest from the health facility in an intention-to-treat analysis.

Sampling strategy and sample size

To estimate the impact of the MWH model based on an intention-to-treat analysis, we aim to select a representative sample of women in our sample frame who delivered a baby in the past 12 months, irrespective of her place of delivery or her use of a MWH. With this strategy, we will also be able to explore the relationship between use of the MWH and location of delivery. As such, we are recruiting a repeated cross-section of 2,400 households at each round for the survey (approximately 60 households per cluster): 1,200 from both intervention and control sites at both baseline (completed in 2016) and endline (planned for 2018), for a total study sample of 4,800 households (Table 3).

Table 3: Total sample size for evaluation

Evaluation Activity	*Intervention Sites*	*Comparison Sites*	*Households per Site*	*X2 Observations (baseline & endline)*	*Total*
Household Survey	20	20	60	2	4800
*In-depth Interview**	*20*	*20*	*6*	*2*	480
TOTAL PARTICIPANTS FOR ALL EVALUATION ACTIVITIES:					**4800**
**Note that IDIs are a subset of the total household survey population selected for more in-depth information and are therefore NOT factored in as additional human subject participants in the total sample size for this study.*					

After accounting for the clustered sampling design (ICC estimated at 0.04 based on previous work [33–35]), and assuming an alpha of .05, this sample will provide us with 80% power to detect a minimum 10 percentage point difference in the anticipated impact of the MWH intervention on the primary outcome of facility delivery, a programmatically meaningful difference. We recruited a sample of 240 women for the IDIs (randomly selecting 10% of the household sample) at baseline, and will recruit another 240 at endline.

For peer review only - http://bmjopen.bmj.com/site/about/guidelines.xhtml

Participant recruitment

For the purposes of this evaluation, a household is defined as a group of people who regularly cook together. Inclusion criteria for the household survey are:

- Household with someone who has delivered a baby within the past 12 months, irrespective of maternal or infant vital status
- Participant must be age 15 or older. If age 15-17, a legal guardian must be available for consent.
- Proxy participant (if woman deceased) must be over the age of 18
- Resident of the village identified for sampling (≥10 km from the facility)

To select a sample representative of women living at least 10km from our health facility, we employ multi-stage random sampling procedures (Figure 3). We begin the first stage of sampling by visiting every village within the catchment area of each study site, informing the local village leader of the purpose of the study and taking the GPS coordinates from the approximate geographical "center" of the village. We input these GPS coordinates into ArcGIS® Online (Esri, Redlands, CA) and use the line creation tool to draw the most direct route along the roads and paths visible on the World Imagery basemap between each village center and their associated health facility. We then use this network of roads to calculate the distance of each village to the health facility and develop a sampling frame of all villages within each catchment area located more than 10km from the health facility (rounding up from 9.5km). We then randomly select a sample of 10 villages from each catchment area with probability proportional to population size. We list every eligible village within a catchment area in Microsoft Excel® along with the total population of the village. We assign a series of numbers to each village, corresponding to the population size (i.e. if village 1 had 30 people, 1-30; village 2 had 20 inhabitants, 31-50), and use the random number generator function to select the villages in each catchment area.

For peer review only - http://bmjopen.bmj.com/site/about/guidelines.xhtml

Second, we work with community volunteers and village leaders to list all households within the selected villages that have a woman who had a delivery in the last year. We randomly order them by rolling a dice twice, first for a random start and then for a random skip until all households are ordered. We visit each household in that order and confirm their eligibility for study participation. We continue down the list until six eligible household in each village are identified. We select additional villages and additional households if necessary to reach our sample of 2,400 households per round. This process assumes that the health facility staff are able to accurately and completely identify all villages within their catchment area.

INSERT FIGURE 3

The study team and community volunteers introduce the study to potential participants and request permission from the household head or most senior woman in the household to screen for eligibility. If household eligibility is confirmed, the study team proceeds with the informed voluntary consent process with the household head or senior woman. Once informed consent is obtained and documented from the household head or senior woman, the enumerator records the geo-location of the household and commences the interview or schedules a later appointment. The household head or senior woman responds to the first part of the survey for approximately 15 minutes, enumerating all of the people in the household in a table that captures demographics as well as recent deliveries and delivery outcomes.

Upon completion of the household demographics and enumeration, an eligible woman was selected to respond to the remainder of the survey. If more than one women in the household had delivered a baby in the past 12 months, the electronic data capture system is programmed to randomly select one eligible woman to respond to the remainder of the survey. The selected woman then

For peer review only - http://bmjopen.bmj.com/site/about/guidelines.xhtml

consents separately, enrolls in the study, and completes the HHS in a private space where she feels comfortable. Completion of the HHS takes approximately 45 minutes.

Of the woman participants, 10% are randomly selected to participate in a 30-minute IDI immediately following the survey. IDI participants can take a short break after the HHS, or reschedule if more convenient. The household-level sampling procedures described here have been conducted at baseline (2016) and will be conducted at endline (2018) with a new cross-sectional sample of households and women within the households. Therefore, the same households are not followed over time.

Patient and public involvement

The development of the research question and outcome measures were informed by key stakeholders and patients' experience and preferences derived from free list responses, key informant interviews, and focus group discussions conducted during the formative evaluation [24–26]. Input from key stakeholders and community members helped to ensure that the design of the intervention would be responsive to community standards of acceptability of factors such as safety and comfort, and a feasible option to increase facility deliveries. Patients were not involved in study design, recruitment, and or conduct of the trial. Given the nature of the intervention, there was limited potential burden on patients and therefore, the burden of the randomized controlled trial was not assessed by the patients.

The primary audience for this evaluation is the Government of Zambia, particularly the Ministry of Health, Ministry of Community Development, and the Ministry of Chiefs and Traditional Affairs, which will use the results to inform the development of maternal and child health strategies and policies in Zambia. We have disseminated the baseline findings to key stakeholders internal to Zambia and will disseminate the full study findings after endline. Many of the findings will likely be of broader interest throughout the region and globally where maternal mortality is high, resources are low, and access to

For peer review only - http://bmjopen.bmj.com/site/about/guidelines.xhtml

facility-based delivery remains an issue. As such, results of this evaluation will be disseminated as widely as possible through open-access journals, websites, and international conferences.

Procedures

Data collection

At baseline and endline, a local team of enumerators literate in the appropriate local language(s) and in English are trained in qualitative and quantitative research methods and human subjects' protection. Surveys are designed in SurveyCTO Collect software (Version 2.212; Dobility, Inc.) and are captured electronically using encrypted tablets. The IDIs are digitally captured on audio recorders. Enumerators explain the tablet system to all participants and explain the digital audio recorders to those selected for IDIs.

Several checks assure the quality of collected survey data. First, enumerators participate in an extensive 5-day training. Second, the enumerators are overseen by data collection team leads with greater experience in data collection fieldwork. Team leads are overseen by a field supervisor. Team leads and the field supervisors review surveys for accuracy and completeness nightly. Third, field supervisors randomly select a 5% subsample of households to be audited; the auditor revisits these households and repeats a subset of survey questions that are checked for reliability. Fourth, the field supervisors conduct a short nightly debrief with the data leads who each oversee three other data collectors and are responsible for conducting the IDIs. Debriefs cover the following topics: field challenges, sampling, total surveys conducted, and IDIs. Lastly, quantitative data are encrypted, uploaded and transferred nightly to the data analysis team where progress is reviewed in real time. On a nightly basis, qualitative data are removed from the recorders, saved on a password-protected computer, and tracked nightly.

Data management

For peer review only - http://bmjopen.bmj.com/site/about/guidelines.xhtml

Survey data are captured on tablets and saved to the internal memory. During each round of data collection, each evening, a data team supervisor reviews the survey and encrypts it so survey data are no longer accessible on the tablet. The supervisor uploads encrypted data nightly during the collection period to a secure server administered by SurveyCTO (Version 2.212; Dobility, Inc.). The evaluation team downloads the encrypted data using the SurveyCTO Client software (Version 2.212; Dobility, Inc.), and decrypts the data using a decryption key generated by the research team.

The evaluation team oversees data entry, management, and storage for qualitative data. All IDIs are translated into English and transcribed verbatim. Digital recorders and paper copies of written notes are kept in a locked cabinet until transcriptions are checked for accuracy and completeness, at which point audio files are deleted and notes are shredded. The electronic transcriptions do not contain identifying information, only a study ID number linked to the quantitative survey. A separate linking file for the quantitative and qualitative data is password protected and only accessible to the study team.

For peer review only

Data analysis

The primary independent variable of interest is assignment to the intervention. For the analysis, we will compare baseline characteristics between the intervention and control groups to assess balance. We collect data on potential confounders to increase precision, analyze heterogeneity, and, if necessary, control for any potential imbalance between the groups.

The primary dependent variable is the probability of facility delivery for most recent birth, based on self-report by mothers. Secondary outcomes include:

- Use of MWHs for antenatal care, delivery or postnatal services
- Delivery by caesarean section
- Maternal death
- Neonatal death

For peer review only - http://bmjopen.bmj.com/site/about/guidelines.xhtml

We initially considered other secondary morbidity outcomes, but because the data were self-reported and asked about experience up to 12 months before, there were limitations to what can reasonably be asked without introducing major recall bias. The survey captures additional indicators of complications including IV antibiotics, blood transfusions, and referral to CEmONC, but we have limited secondary outcomes to those most likely to be clearly remembered.

All quantitative analyses will be conducted in SAS v9.4 (SAS, Cary, NC). Our quantitative analytic plan is threefold, yielding descriptive, bivariate and multivariate statistics. First, we will describe the study sample, stratifying by intervention and control group and testing for differences between the groups. Second, we will estimate differences between the groups for primary and secondary outcomes, controlling for a set of baseline demographics. Categorical variables will be compared between the groups using a chi-squared test when cell sizes are sufficient or Fisher's exact test when the cell sizes are small; continuous variables will be compared using t-tests if normally distributed or non-parametric Wilcoxon rank sum tests if the distribution is non-normal. Third, we will fit several regression models to estimate the impact of the intervention on the primary and secondary outcomes, adjusting for baseline values, assignment matching variables, and any imbalanced covariates. To control for the phased timing of implementation, we include a variable in our main models that captures the month the home opened.

All qualitative data will be analyzed in Nvivo 10 © software (QSR International Pty Ltd.). We will conduct a content analysis of the IDI transcripts. Coding themes have been identified *a priori*. Additional themes will be included as they emerge. We will triangulate findings with the quantitative data to identify consistencies, inconsistencies or additional themes to be explored. We will use the themes developed during the baseline analysis to analyze the endline data and identify any new themes as they emerge.

For peer review only

To systematically assess confounders and the risk of bias at the pre-intervention phase, intervention phase, and post-intervention phase, we will use the ROBINS-I tool.[36] Guided by this tool, we will transparently report threats to validity of this quasi-experimental study during analysis, interpretation and dissemination. Results for the primary and each secondary evaluation question will be presented.

ETHICS

Ethics approval and consent to participate

Ethical review boards

Prior to participant enrollment, ethical approvals were obtained from the Boston University Institutional Review Board (IRB), University of Michigan IRB (for a de-identified dataset only), and the ERES Converge Research IRB, a private local ethics board in Zambia. We also obtained official approval to proceed with the study from the Zambia National Health Research Authority, which is responsible for oversight of all research conducted in the country. Adverse events, unanticipated problems and any protocol changes will be reported to the IRBs and the Zambia National Health Research Authority per their guidelines, and all investigators will be informed.

Potential risks and protections

This study poses minimal risk to study participants and several steps were taken to minimize risk and burden. To reduce the risk of disclosure of personal or sensitive information enumerators are trained to stop participants from disclosing information that is too sensitive. Participation may cause some discomfort from answering certain questions, particularly if the maternal or neonatal health outcomes were adverse. Enumerators are trained to minimize any potential discomfort or harm to all participants during all study activities to the greatest extent possible. We minimize any waiting by participants by

For peer review only - http://bmjopen.bmj.com/site/about/guidelines.xhtml

scheduling meetings during times convenient to participants and interviews are kept to as short of time as possible taking breaks if necessary.

Potential benefits

There are no direct individual benefits to participating in the study. The evaluation results will generate evidence on the impact of MWHs on facility delivery for those who live farthest away. Findings will provide insight for policy makers into how, if found to be effective, MWHs can be part of a broader strategy to improve maternal and neonatal health outcomes.

Participant confidentiality

Throughout the study, we take care to ensure the confidentiality of data obtained from study participants. The HHSs and IDIs are carried out in participants' private homes or somewhere the participant feels comfortable. We do not proceed with data collection until we can confirm that the location is acceptable and participants agree that they feel comfortable discussing study topics.

The linking file with identifiable data and basic demographics is stored in a separate file within the tablet system. Upon completion of data collection, all files are stored on a secure server during data analysis and report writing. Only BU/RTC investigators have access to identifiable data. All analyses by study partners are conducted on de-identified datasets per IRB approvals. Analyses are presented in aggregate format in technical reports to stakeholders and in manuscripts submitted for publication in scientific journals. Under no circumstances do organizations or individuals have access to the participant's individual demographic information and potential identifying information (job title, age range, sex, and village). As explained above, the qualitative data are de-identified, with basic demographics only.

For peer review only

Informed consent

Prior to any data collection, we discuss the purpose of the study with local leaders so that the study activities are clearly understood. If a household is eligible, the study team proceeds with the informed voluntary consent process from the household head or the most senior woman in the household, introducing themselves, the purpose of the study, and explaining what we are asking of them in terms of participation, the risks and benefits, the right to withdraw without penalty at any time, that their information will be kept in a safe location, and that their answers will not be linked to their names. Participants are informed that the alternative is to not participate in the study. The study team slowly and clearly asks for consent to participate. If a selected household participant declines participation, the next household on the randomly ordered list of eligible households is contacted. If a household head or senior woman consents to participating, the study team documents written informed consent and proceeds with the interview. In addition to the household head or a senior woman, using the same process we also consent the woman selected from within the household to respond to the survey; in some cases, this may be the same person. A maximum of two individuals are consented per household.

We anticipate about 15% of the sample in each round to be between 15-17 years of age. In Zambia, 'emancipated minors' can enroll if they provide assent and their guardian or husband also provides consent. If a woman's husband is 18 or older, then he can provide informed consent on behalf of his wife; however, if he is also under 18 years old, then her legal guardian must provide consent. If under 18, the research team will allow the woman to first determine if she wishes to join the study (assent is provided) and then obtain consent by the guardian or husband. Thus, the individual's wishes are protected and she can determine if she wishes to be part of the study.

All informed consent or assent/consent is documented with a signature; in the event a participant cannot write, a witness signs the informed consent. A participant retains a copy of the

For peer review only

For peer review only - http://bmjopen.bmj.com/site/about/guidelines.xhtml

informed consent form. The informed consent and assent processes are always conducted in the language most preferred by the participant.

Costs and payments

For all activities, the participants volunteer only the time taken to complete this survey. There is no cash payment provided to participants for any portion of the study. Participants receive pieces of fabric as small tokens of appreciation in recognition of their time and opportunity costs, in line with local IRB procedures.

Limitations

This study has several limitations. First, half of study clusters could not be randomly assigned to either the intervention or control group due to political constraints and concern by the government about community fatigue. The selection bias resulting from the different assignment strategies is partially mitigated by ensuring comparison sites are matched on the same criteria as the other sites. Additionally, because one partner's comparison sites include existing MWHs as part of standard of care, and the other partner excluded sites with existing MWHs, we will analyze the full sample as a quasi-experimental CBA study and we will estimate the impact in both the non-randomized and randomized subsamples to assess potential bias introduced by non-random assignment and the differences in comparison site selection. Second, we limited household eligibility to those living at least 10 km from the health facilities and will not be able to assess impact on women living nearer to facilities. However, remote women are the primary target of the MWH model and stand to benefit the most from the intervention. To manage this limitation, in separate process evaluation protocols, each partner is collecting facility-based data to understand any changes in demographics among those utilizing facilities for delivery using agreed upon indicators and data sources. Lastly, because there are two implementing partners, there is a risk that the MWH model will be implemented differently across the sites. To

For peer review only

mitigate this risk, we have developed and agreed upon the precise elements of the MWH model based on both partners' formative research [24–26] and will be assessing implementation fidelity using harmonized tools in the companion process evaluation protocols.

REFERENCES

1 The United Nations. Goal 3 .:. Sustainable Development Knowledge Platform. https://sustainabledevelopment.un.org/sdg3 (accessed 2 Feb 2018).

2 Central Statistical Office (CSO) Zambia, [Ministry of Health (MOH) [Zambia], ICF International. Zambia Demographic and Health Survey 2013-14. Rockville, Maryland, USA: 2014.

3 UN General Assembly. Resolution adopted by the General Assembly on 25 September 2015 70/1. Transforming our world: the 2030 Agenda for Sustainable Development. 2030. http://www.un.org/en/development/desa/population/migration/generalassembly/docs/globalcompact/A_RES_70_1_E.pdf (accessed 17 Jan 2018).

4 Republic of Zambia Ministry of Health. National Health Strategic Plan 2011 - 2015. 2011;:119.

5 Republic of Zambia Ministry of Health. Roadmap for Accelerating Reduction of Maternal , Newborn and Child Mortality , 2013-2016. 2016;:2013–6.

6 Gabrysch S, Cousens S, Cox J, *et al.* The influence of distance and level of care on delivery place in rural Zambia: a study of linked national data in a geographic information system. *PLoS Med* 2011;**8**. doi:10.1371/journal.pmed.1000394

7 Gabrysch S, Campbell OM, Lawn J, *et al.* Still too far to walk: Literature review of the determinants of delivery service use. *BMC Pregnancy Childbirth* 2009;**9**:34. doi:10.1186/1471-2393-9-34

8 Lohela TJ, Campbell OMR, Gabrysch S, *et al.* Distance to Care, Facility Delivery and Early Neonatal Mortality in Malawi and Zambia. *PLoS One* 2012;**7**:e52110. doi:10.1371/journal.pone.0052110

9 Sialubanje C, Massar K, Hamer DH, *et al.* Understanding the psychosocial and environmental factors and barriers affecting utilization of maternal healthcare services in Kalomo, Zambia: A qualitative study. *Health Educ Res* 2014;**29**:521–32. doi:10.1093/her/cyu011

10 Stekelenburg J, Van Lonkhuijzen L, Spaans W, *et al.* Maternity waiting homes in rural districts in Africa: A cornerstone of safe motherhood? *Curr Womens Health Rev* 2006;**2**:235–8. doi:10.2174/157340406778699914

11 Lori JR, Wadsworth AC, Munro ML, *et al.* Promoting access: The use of maternity waiting homes to achieve safe motherhood. *Midwifery* 2013;**29**:1095–102. doi:10.1016/j.midw.2013.07.020

12 Kelly J, Kohls E, Poovan P, *et al.* The role of a maternity waiting area (MWA) in reducing maternal mortality and stillbirths in high-risk women in rural Ethiopia. *BJOG* 2010;**117**:1377–83. doi:10.1111/j.1471-0528.2010.02669.x

For peer review only

For peer review only - http://bmjopen.bmj.com/site/about/guidelines.xhtml

13 Henry EG, Semrau K, Hamer DH, *et al.* The influence of quality maternity waiting homes on utilization of facilities for delivery in rural Zambia. *Reprod Health* 2017;**14**:68. doi:10.1186/s12978-017-0328-z

14 Fogliati P, Straneo M, Mangi S, *et al.* A new use for an old tool: maternity waiting homes to improve equity in rural childbirth care. Results from a cross-sectional hospital and community survey in Tanzania. *Health Policy Plan* 2017;**32**:1354–60. doi:10.1093/heapol/czx100

15 García Prado A, Cortez R. Maternity waiting homes and institutional birth in Nicaragua: policy options and strategic implications. *Int J Health Plann Manage* 2012;**27**:150–66. doi:10.1002/hpm.1107

16 Chandramohan D, Cutts F, Millard P. The effect of stay in a maternity waiting home on perinatal mortality in rural Zimbabwe. *J Trop Med Hyg* 1995;**98**:261–7.http://www.ncbi.nlm.nih.gov/pubmed/7636923 (accessed 12 Dec 2016).

17 Chandramohan D, Cutts F, Chandra R. Effects of a maternity waiting home on adverse maternal outcomes and the validity of antenatal risk screening. *Int J Gynecol Obstet* 1994;**46**:279–84. doi:10.1016/0020-7292(94)90406-5

18 Gaym A, Pearson L, Soe KWW. Maternity waiting homes in Ethiopia--three decades experience. *Ethiop Med J* 2012;**50**:209–19.

19 Van Lonkhuijzen L, Stegeman M, Nyirongo R, *et al.* Use of Maternity Waiting Home in Rural Zambia. *Afr J Reprod Health* 2003;**7**:32–6. doi:10.1108/10650750910961929

20 Andemichael G, Haile B, Kosia A, *et al.* Maternity waiting homes: A panacea for maternal/neonatal conundrums in Eritrea. *J Eritrean Med Assoc* 2009;**4**:18–21. doi:10.4314/jema.v4i1.52112

21 van Lonkhuijzen L, Stekelenburg J, van Roosmalen J. Maternity waiting facilities for improving maternal and neonatal outcome in low-resource countries. *Cochrane database Syst Rev* 2012;**10**:CD006759. doi:10.1002/14651858.CD006759.pub3

22 Wild K, Barclay L, Kelly P, *et al.* The tyranny of distance: maternity waiting homes and access to birthing facilities in rural Timor-Leste. *Bull World Health Organ* 2012;**90**:97–103. doi:10.2471/BLT.11.088955

23 Ministry of Health (MOH) [Zambia]. Zambia National Health Strategic Plan 2017-2021. 2017.

24 Chibuye PS, Bazant ES, Wallon M, *et al.* Experiences with and expectations of maternity waiting homes in Luapula Province, Zambia: a mixed–methods, cross-sectional study with women, community groups and stakeholders. *BMC Pregnancy Childbirth* 2018;**18**:42. doi:10.1186/s12884-017-1649-1

25 Lori JR, Munro-Kramer ML, Mdluli EA, *et al.* Developing a community driven sustainable model of maternity waiting homes for rural Zambia. *Midwifery* 2016;**41**:89–95. doi:10.1016/j.midw.2016.08.005

26 Scott NA, Vian T, Kaiser JL, *et al.* Listening to the community: Using formative research to strengthen maternity waiting homes in Zambia. *PLoS One* 2018;**13**. doi:https://doi.org/10.1371/journal.pone.0194535

27 Central Statistical Office (CSO) Zambia. 2010 Census of Population and Housing - Southern Province Analytical Report. Lusaka: 2014. http://web0.psa.gov.ph/statistics/census/population-and-housing

For peer review only - http://bmjopen.bmj.com/site/about/guidelines.xhtml

28 Central Statistical Office (CSO) Zambia. 2010 Census of Population and Housing - Eastern Province Analytical Report. Lusaka: 2014. https://www.zamstats.gov.zm/phocadownload/2010_Census/2010_Census_Analytical_Reports/Eastern Province Analytical Report - 2010 Census.pdf (accessed 19 Jan 2018).
29 Central Statistical Office (CSO) Zambia. Zambia 2010 Census of Population and Housing Population Summary Report. Lusaka: 2012. http://www.mcaz.gov.zm/wp-content/uploads/2014/10/2010-Census-of-Population-Summary-Report.pdf (accessed 19 Jan 2018).
30 Zambian Ministry of Health. The 2012 List of Health Facilities in Zambia: Preliminary Report, v15. 2013;:252.
31 SMGL. Saving Mothers Giving Life - Home. 2016.https://www.savingmothersgivinglife.org/ (accessed 16 Nov 2016).
32 Bernard HR. *Social Research Methods Qualitative and Quantitative Approaches*. 2nd ed. USA: : SAGE Publications, Inc 2013.
33 Hamer DH, Herlihy JM, Musokotwane K, *et al.* Engagement of the Community, Traditional Leaders, and Public Health System in the Design and Implementation of a Large Community-Based, Cluster-Randomized Trial of Umbilical Cord Care in Zambia. *Am J Trop Med Hyg* 2015;**92**:666–72. doi:10.4269/ajtmh.14-0218
34 Semrau KEA, Herlihy J, Grogan C, *et al.* Effectiveness of 4% chlorhexidine umbilical cord care on neonatal mortality in Southern Province, Zambia (ZamCAT): a cluster-randomised controlled trial. *Lancet Glob Heal* 2016;**4**:e827–36. doi:10.1016/S2214-109X(16)30215-7
35 Pagel C, Prost A, Lewycka S, *et al.* Intracluster correlation coefficients and coefficients of variation for perinatal outcomes from five cluster-randomised controlled trials in low and middle-income countries: results and methodological implications. *Trials* 2011;**12**:151. doi:10.1186/1745-6215-12-151
36 Sterne JA, Hernán MA, Reeves BC, *et al.* ROBINS-I: a tool for assessing risk of bias in non-randomised studies of interventions. *BMJ* 2016;**355**:i4919. doi:10.1136/BMJ.I4919

AUTHORS STATEMENT

NS led the scientific design, implementation, and was primarily responsible for drafting this manuscript. JK contributed to the development of the protocol, led the development of the study sample, coordinated data collection, and contributed to revisions of the manuscript. TV and RB contributed to the revisions and science of the protocol and data collection instruments. RF contributed to the development of the protocol, implemented the electronic data capture system, and contributed to the revisions of this manuscript. TN, GB, CB, and JL provided feedback on the protocol and reviewed and edited the final manuscript. DH provided scientific support, technical input into the survey design and

For peer review only - http://bmjopen.bmj.com/site/about/guidelines.xhtml

critically reviewed and edited the final manuscript. PR helped conceptualize the scientific design of the study, provided technical input into the survey design, sampling approach, and critically reviewed and edited the final manuscript. All authors read and approved the final version of the manuscript.

FUNDING

This program was developed and is being implemented in collaboration with *MSD for Mothers*, MSD's 10-year, $500 million initiative to help create a world where no woman dies giving life. *MSD for Mothers* is an initiative of Merck & Co., Inc., Kenilworth, N.J., U.S.A. (MRK 1846-06500.COL). The development of this article was additionally supported in part by the Bill & Melinda Gates Foundation (OPP1130329) https://www.gatesfoundation.org/How-We-Work/Quick-Links/Grants-Database/Grants/2015/07/OPP1130329 and The ELMA Foundation (ELMA-15-F0017) http://www.elmaphilanthropies.org/the-elma-foundation/. The funders had no role in study design, data collection and analysis, decision to publish, or preparation of the manuscript. The content is solely the responsibility of the authors and does not reflect positions or policies of MSD, the Bill & Melinda Gates Foundation or The ELMA Foundation.

w only

CONFLICTS OF INTEREST STATEMENT

The authors declare that they have no competing interests.

FIGURE LEGEND

Figure 1. Minimum Maternity Waiting Home Model in the Maternity Home Alliance for Intervention Sites (n=20).

Figure 2. **Map of the Maternity Home Alliance Intervention and Control Study Sites by Partner**

Figure 3. Multi-stage Random Sampling Strategy for Baseline and Endline

For peer review only - http://bmjopen.bmj.com/site/about/guidelines.xhtml

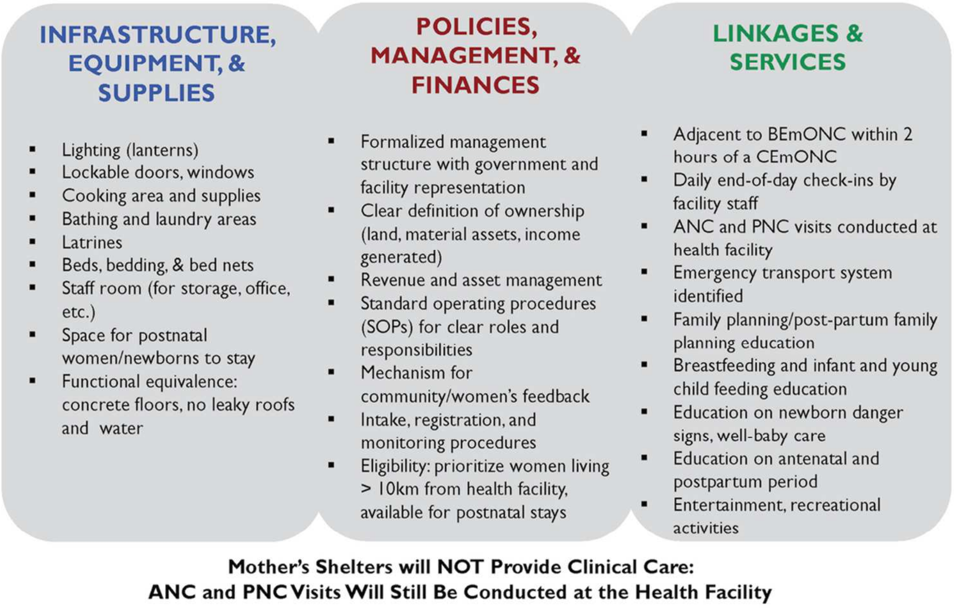

Figure 1. Minimum Maternity Waiting Home Model in the Maternity Home Alliance for Intervention Sites (n=20).

101x66mm (300 x 300 DPI)

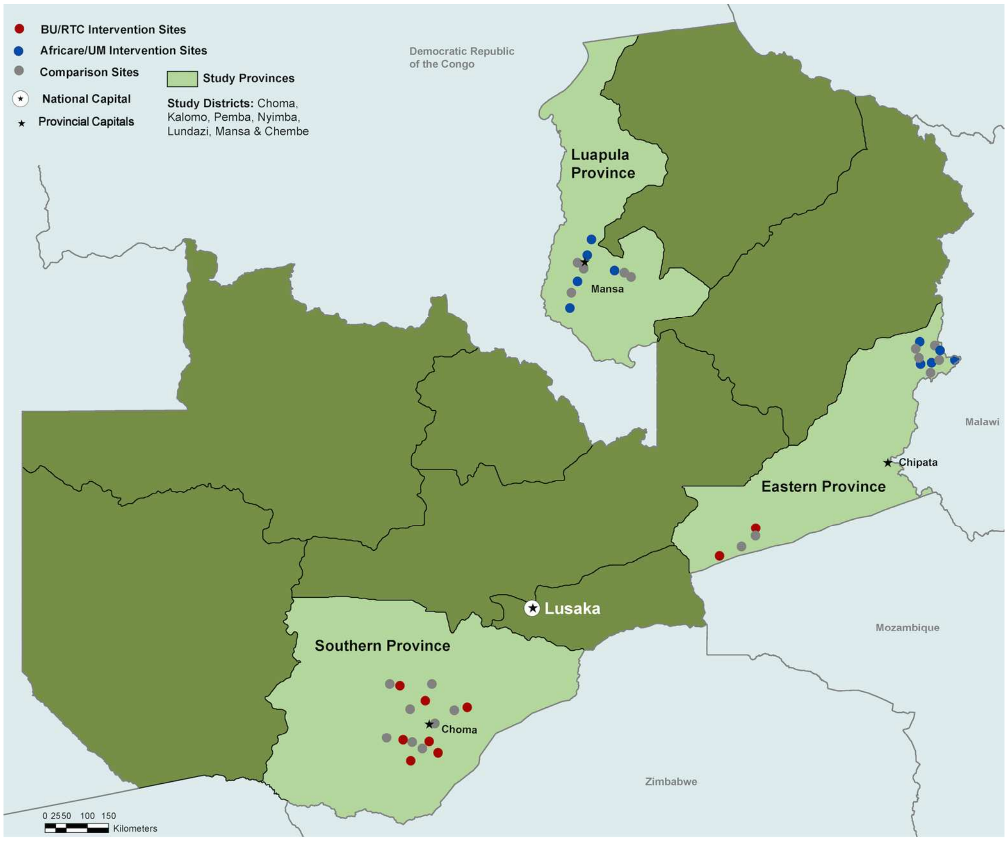

Figure 2. Map of the Maternity Home Alliance Intervention and Control Study Sites by Partner

173x144mm (300 x 300 DPI)

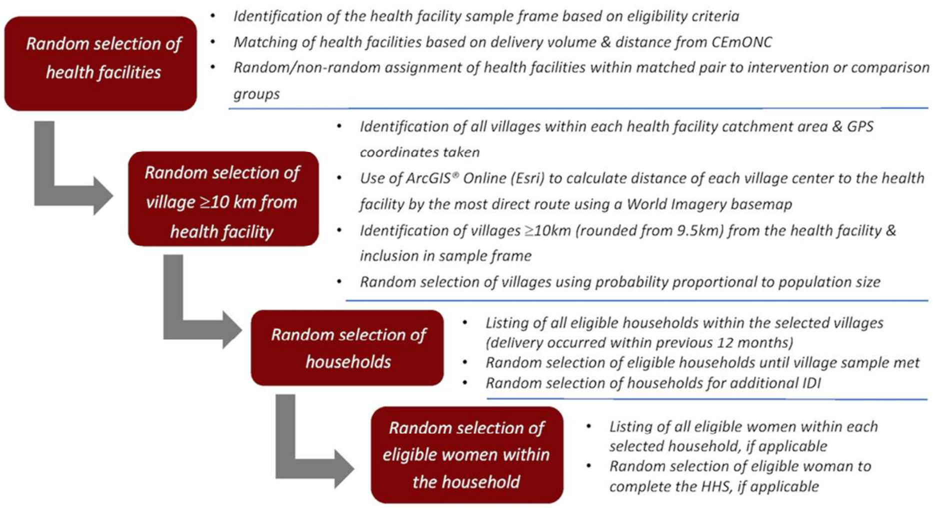

Figure 3. Multi-stage Random Sampling Strategy for Baseline and Endline

97x54mm (300 x 300 DPI)

STANDARD PROTOCOL ITEMS: RECOMMENDATIONS FOR INTERVENTIONAL TRIALS

SPIRIT 2013 Checklist: Recommended items to address in a clinical trial protocol and related documents*

Section/item	Item No	Description	Addressed on page number
**Administrative information**			
Title	1	Descriptive title identifying the study design, population, interventions, and, if applicable, trial acronym	_____1________
Trial registration	2a	Trial identifier and registry name. If not yet registered, name of intended registry	_3,clinicaltrials.gov
	2b	All items from the World Health Organization Trial Registration Data Set	1-23 and on clinicaltrials.gov
Protocol version	3	Date and version identifier	______na_____
Funding	4	Sources and types of financial, material, and other support	______23_____
Roles and responsibilities	5a	Names, affiliations, and roles of protocol contributors	______1_______
	5b	Name and contact information for the trial sponsor	______23______
	5c	Role of study sponsor and funders, if any, in study design; collection, management, analysis, and interpretation of data; writing of the report; and the decision to submit the report for publication, including whether they will have ultimate authority over any of these activities	______23_______

For peer review only

For peer review only - http://bmjopen.bmj.com/site/about/guidelines.xhtml

	5d	Composition, roles, and responsibilities of the coordinating centre, steering committee, endpoint adjudication committee, data management team, and other individuals or groups overseeing the trial, if applicable (see Item 21a for data monitoring committee)	____na_________
**Introduction**			
Background and rationale	6a	Description of research question and justification for undertaking the trial, including summary of relevant studies (published and unpublished) examining benefits and harms for each intervention	_____5,6______
	6b	Explanation for choice of comparators	______8_______
Objectives	7	Specific objectives or hypotheses	_____5,6_______
Trial design	8	Description of trial design including type of trial (eg, parallel group, crossover, factorial, single group), allocation ratio, and framework (eg, superiority, equivalence, noninferiority, exploratory)	_____8________
**Methods: Participants, interventions, and outcomes**			
Study setting	9	Description of study settings (eg, community clinic, academic hospital) and list of countries where data will be collected. Reference to where list of study sites can be obtained	_____7________
Eligibility criteria	10	Inclusion and exclusion criteria for participants. If applicable, eligibility criteria for study centres and individuals who will perform the interventions (eg, surgeons, psychotherapists)	_____8,9,12____
Interventions	11a	Interventions for each group with sufficient detail to allow replication, including how and when they will be administered	______6_______
	11b	Criteria for discontinuing or modifying allocated interventions for a given trial participant (eg, drug dose change in response to harms, participant request, or improving/worsening disease)	______na_______
	11c	Strategies to improve adherence to intervention protocols, and any procedures for monitoring adherence (eg, drug tablet return, laboratory tests)	_____na________
	11d	Relevant concomitant care and interventions that are permitted or prohibited during the trial	______na_______

For peer review only - http://bmjopen.bmj.com/site/about/guidelines.xhtml

Outcomes	12	Primary, secondary, and other outcomes, including the specific measurement variable (eg, systolic blood pressure), analysis metric (eg, change from baseline, final value, time to event), method of aggregation (eg, median, proportion), and time point for each outcome. Explanation of the clinical relevance of chosen efficacy and harm outcomes is strongly recommended	____6,7,16______
Participant timeline	13	Time schedule of enrolment, interventions (including any run-ins and washouts), assessments, and visits for participants. A schematic diagram is highly recommended (see Figure)	_____11_______
Sample size	14	Estimated number of participants needed to achieve study objectives and how it was determined, including clinical and statistical assumptions supporting any sample size calculations	_____11________
Recruitment	15	Strategies for achieving adequate participant enrolment to reach target sample size	_____13________
**Methods: Assignment of interventions (for controlled trials)**			
Allocation:			
Sequence generation	16a	Method of generating the allocation sequence (eg, computer-generated random numbers), and list of any factors for stratification. To reduce predictability of a random sequence, details of any planned restriction (eg, blocking) should be provided in a separate document that is unavailable to those who enrol participants or assign interventions	____9, 12, 13, __
Allocation concealment mechanism	16b	Mechanism of implementing the allocation sequence (eg, central telephone; sequentially numbered, opaque, sealed envelopes), describing any steps to conceal the sequence until interventions are assigned	______na_______
Implementation	16c	Who will generate the allocation sequence, who will enrol participants, and who will assign participants to interventions	_____na________
Blinding (masking)	17a	Who will be blinded after assignment to interventions (eg, trial participants, care providers, outcome assessors, data analysts), and how	______na_______
	17b	If blinded, circumstances under which unblinding is permissible, and procedure for revealing a participant's allocated intervention during the trial	______na_______
**Methods: Data collection, management, and analysis**			

For peer review only - http://bmjopen.bmj.com/site/about/guidelines.xhtml

Data collection methods	18a	Plans for assessment and collection of outcome, baseline, and other trial data, including any related processes to promote data quality (eg, duplicate measurements, training of assessors) and a description of study instruments (eg, questionnaires, laboratory tests) along with their reliability and validity, if known. Reference to where data collection forms can be found, if not in the protocol	____6-14_______
	18b	Plans to promote participant retention and complete follow-up, including list of any outcome data to be collected for participants who discontinue or deviate from intervention protocols	_____na________
Data management	19	Plans for data entry, coding, security, and storage, including any related processes to promote data quality (eg, double data entry; range checks for data values). Reference to where details of data management procedures can be found, if not in the protocol	____14, 15______
Statistical methods	20a	Statistical methods for analysing primary and secondary outcomes. Reference to where other details of the statistical analysis plan can be found, if not in the protocol	_____15, 16_____
	20b	Methods for any additional analyses (eg, subgroup and adjusted analyses)	______16_______
	20c	Definition of analysis population relating to protocol non-adherence (eg, as randomised analysis), and any statistical methods to handle missing data (eg, multiple imputation)	______na_______
**Methods: Monitoring**			
Data monitoring	21a	Composition of data monitoring committee (DMC); summary of its role and reporting structure; statement of whether it is independent from the sponsor and competing interests; and reference to where further details about its charter can be found, if not in the protocol. Alternatively, an explanation of why a DMC is not needed	_____na________
	21b	Description of any interim analyses and stopping guidelines, including who will have access to these interim results and make the final decision to terminate the trial	______na_______
Harms	22	Plans for collecting, assessing, reporting, and managing solicited and spontaneously reported adverse events and other unintended effects of trial interventions or trial conduct	______16, 17____
Auditing	23	Frequency and procedures for auditing trial conduct, if any, and whether the process will be independent from investigators and the sponsor	______na_______
**Ethics and dissemination**			

For peer review only - http://bmjopen.bmj.com/site/about/guidelines.xhtml

Research ethics approval	24	Plans for seeking research ethics committee/institutional review board (REC/IRB) approval	______16_______
Protocol amendments	25	Plans for communicating important protocol modifications (eg, changes to eligibility criteria, outcomes, analyses) to relevant parties (eg, investigators, REC/IRBs, trial participants, trial registries, journals, regulators)	______16, 17____
Consent or assent	26a	Who will obtain informed consent or assent from potential trial participants or authorised surrogates, and how (see Item 32)	____18, 19______
	26b	Additional consent provisions for collection and use of participant data and biological specimens in ancillary studies, if applicable	_____na________
Confidentiality	27	How personal information about potential and enrolled participants will be collected, shared, and maintained in order to protect confidentiality before, during, and after the trial	____17, 18______
Declaration of interests	28	Financial and other competing interests for principal investigators for the overall trial and each study site	________23______
Access to data	29	Statement of who will have access to the final trial dataset, and disclosure of contractual agreements that limit such access for investigators	_____18________
Ancillary and post-trial care	30	Provisions, if any, for ancillary and post-trial care, and for compensation to those who suffer harm from trial participation	_____na________
Dissemination policy	31a	Plans for investigators and sponsor to communicate trial results to participants, healthcare professionals, the public, and other relevant groups (eg, via publication, reporting in results databases, or other data sharing arrangements), including any publication restrictions	______19_______
	31b	Authorship eligibility guidelines and any intended use of professional writers	_____22, 23_____
	31c	Plans, if any, for granting public access to the full protocol, participant-level dataset, and statistical code	______na_______
**Appendices**			
Informed consent materials	32	Model consent form and other related documentation given to participants and authorised surrogates	_Can be provided upon request__

For peer review only - http://bmjopen.bmj.com/site/about/guidelines.xhtml

Biological specimens	33	Plans for collection, laboratory evaluation, and storage of biological specimens for genetic or molecular analysis in the current trial and for future use in ancillary studies, if applicable	_____na________

*It is strongly recommended that this checklist be read in conjunction with the SPIRIT 2013 Explanation & Elaboration for important clarification on the items. Amendments to the protocol should be tracked and dated. The SPIRIT checklist is copyrighted by the SPIRIT Group under the Creative Commons "Attribution-NonCommercial-NoDerivs 3.0 Unported" license.

For peer review only

For peer review only - http://bmjopen.bmj.com/site/about/guidelines.xhtml
